# Cumulative Evidence for Relationships Between Multiple Variants in the TERT and CLPTM1L Region and Risk of Cancer and Non-Cancer Disease

**DOI:** 10.3389/fonc.2022.946039

**Published:** 2022-06-30

**Authors:** Jie Tian, Yan Wang, Yingxian Dong, Junke Chang, Yongming Wu, Shuai Chang, Guowei Che

**Affiliations:** ^1^ Department of Thoracic Surgery, West China Hospital, Sichuan University, Chengdu, China; ^2^ Department of Lung Cancer Center, West China Hospital, Sichuan University, Chengdu, China

**Keywords:** *TERT*, *CLPTM1L*, genetic variant, disease, susceptibility

## Abstract

**Background:**

Genetic studies previously reported that variants in *TERT-CLPTM1L* genes were related to susceptibility of cancer and non-cancer diseases. However, conclusions were not always concordant.

**Methods:**

We performed meta-analyses to assess correlations between 23 variants within *TERT-CLPTM1L* region and susceptibility to 12 cancers and 1 non-cancer disease based on data in 109 papers (involving 139,510 cases and 208,530 controls). Two approaches (false-positive report probability test and Venice criteria) were adopted for assessing the cumulative evidence of significant associations. Current study evaluated the potential role of these variants based on data in Encyclopedia of DNA Elements (ENCODE) Project.

**Results:**

Thirteen variants were statistically associated with susceptibility to 11 cancers and 1 non-cancer disease (*p* < 0.05). Besides, 12 variants with eight cancers and one non-cancer disease were rated as strong evidence (rs2736098, rs401681, and rs402710 in bladder cancer; rs2736100, rs2853691, and rs401681 in esophageal cancer; rs10069690 in gastric cancer; rs2736100 and rs2853676 in glioma; rs2242652, rs2736098, rs2736100, rs2853677, rs31489, rs401681, rs402710, rs465498, and rs4975616 in lung cancer; rs2736100 in idiopathic pulmonary fibrosis and myeloproliferative neoplasms; and rs401681 in pancreatic and skin cancer). According to data from ENCODE and other public databases, 12 variants with strong evidence might fall within putative functional regions.

**Conclusions:**

This paper demonstrated that common variants of *TERT-CLPTM1L* genes were related to susceptibility to bladder, esophageal, gastric, lung, pancreatic, and skin cancer, as well as to glioma, myeloproliferative neoplasms, and idiopathic pulmonary fibrosis, and, besides, the crucial function of the *TERT-CLPTM1L* region in the genetic predisposition to human diseases is elucidated.

## Introduction

Cancer as a dominating reason for death is threatening life and health of human being worldwide, with ~19.3 million newly diagnosed tumor patients along with ~10.0 million cancer-associated death cases in 2020 (1). Both environmental and genetic factors lead to cancer occurrence and progression, and 5%– 10% of cancer is resulted from variation in genes (2). Genome-wide association studies (GWAS) and genetic association research have proved multiple single-nucleotide polymorphisms (SNPs) linked with risk of human diseases (3).

The telomerase reverse transcriptase (*TERT*) gene and cleft lip and palate transmembrane 1–like (*CLPTM1L)* gene are mapped on chromosome 5p15.33. Of them, *TERT* gene, encoding the rate-limiting telomerase enzyme for catalysis, exerts a significant influence on maintaining cell immortality, telomere DNA length, and chromosomal stability (4). The protein encoded by *CLPTM1L* is a membrane protein associated with cisplatin resistance, and the overexpression of *CLPTM1L* in cisplatin-sensitive cells causes apoptosis (5). In early 1990s, researchers had made attempts to account for the existing relationships of telomeres, telomerase, aging, and cancer risk (6, 7). Wang et al. first uncovered that a novel variant (*TERT* MNS16A) had an elevated risk of lung cancer in 2003 (8). In 2006, Matsubara et al. first revealed that *TERT* rs2735940 had an elevated risk of coronary artery disease (non-cancer disease) (9). Since then, numerous genetic association studies were conducted to investigate the associations among SNPs of *TERT* and *CLPTM1L* regions with human diseases. In 2008, a GWAS was performed on the Caucasian population, and then it was found that two variants (rs402710 and rs2736100) were featured with a higher susceptibility to lung cancer (10). Subsequently, from a Japanese GWAS, it was seen that rs2736100 increased risk of idiopathic pulmonary fibrosis (11). Apart from idiopathic pulmonary fibrosis, several GWAS showed that rs2736100 could enhance lung cancer and testicular germ cell cancer susceptibility in Caucasians (12, 13) while could decrease the risk of idiopathic interstitial pneumonia and glioma in Caucasians (14, 15) and lung cancer in Asians (16). Moreover, in a GWAS conducted in multiple countries from the European ancestry, rs401681 associated with 75,000 individuals was tested, and then it was discovered that this SNP could elevate susceptibility to lung, urinary bladder, prostate, and basal cell cancer while could decrease cutaneous melanoma susceptibility (17).

Even though numerous genetic association studies investigate the association of variant in *TERT* and *CLPTM1L* regions and cancers or non-cancer disease susceptibility, the conclusions are not always consistent and the functional mechanisms remain unclear. Although, in previous published meta-analysis studies, a single SNP (for example, rs2736100 and rs2736098) with risk of individual cancer (18–20) was investigated, the results were still inconsistent. Besides, a comprehensive research synopsis with systematic functional annotation had not been performed to evaluate the epidemiological evidence of genetic correlations between *TERT* and *CLPTM1L* genes and susceptibility to cancers or non-cancer disease until now. As a result, we conducted meta-analyses to account for the relationships of SNPs in the *TERT-CLPTM1L* genes with cancers or non-cancer disease predisposition, provided the epidemiological evidence for variants with significant associations, and assessed the roles of significant SNPs using information from public databases.

## Material and Methods

This study was conducted by following the Preferred Reporting Items for Systematic Reviews and Meta-Analyses Statement guidelines (**Supplementary Table 1**) and the Human Genome Epidemiology Network for systematic review of genetic association studies (21, 22).

**Figure 1:**
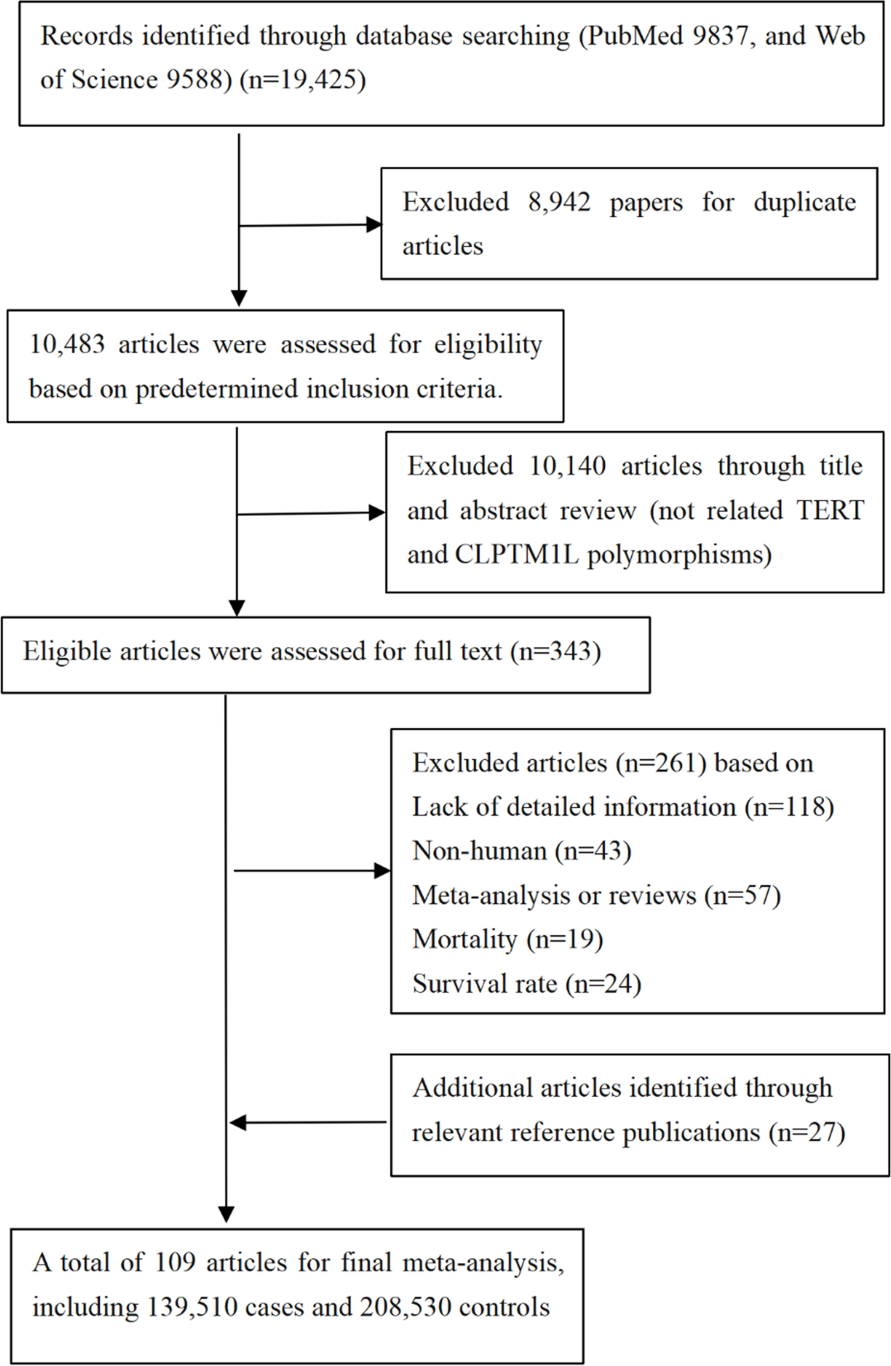
Flow diagram of search strategy and study selection.

Here, papers from PubMed, Web of science, and Embase before 30 Dec 2021 were screened using “{human telomerase reverse transcriptase} OR {hTERT} OR {TERT} OR {CLPTM1L} OR {cleft and palate transmembrane 1 like} OR {TERT-CLPTM1L region} OR {5p15.33}”, and 19,425 citations were identified. Apart from that, additional articles were also collected through examining the relevant references of publications (reviews, meta-analysis studies, etc.). Finally, 109 papers were included in our study (**Figure 1**).

The included criteria were as follows: (1) assessing relationships of variations in the *TERT-CLPTM1L* region with human cancers or non-cancer disease by conducting case-control, cross-sectional, cohort studies, or GWAS; (2) providing details of genotype amount for computing the values of odds ratios (ORs) and 95% confidence intervals (95% CIs); (3) being published or full text in English, whereas the criteria of exclusion are shown as follows: (1) there is no sufficient information (especially genotype amount); (2) the interests is not *TERT-CLPTM1L* region polymorphism; (3) the study is a letter to editors or conference abstract; (iv) the interests are cancer mortality (but not incidence).

### Data Extraction

Two investigators obtained data from eligible papers on their own. Any inconsistencies could be addressed by discussion with the rest of investigators. As for included variants, the information was extracted, as follows: first author, publishing year, the country or region, type of cancer and non-cancer disease, ethnicity, the gene name, the variant, amount of genotype, cases and controls, and minor allele frequency (MAF). Apart from that, ethnicity comprises four parts [Asians (East Asian descent), Caucasians (European descent), Africans (African descent), or others (Indians, Native Hawaiians, Latinos, Hispanics and the mixed, etc.)] following that more than 80% of research subjects were within the abovementioned groups; “overall populations” are composed of two or more. Furthermore, in this study, the data were extracted from publications with the largest individuals and the most comprehensive data if numerous papers had the same or overlapping data.

### Statistical Analysis

*P* < 0.05 (two-sided) indicated the significance threshold, which was computed with the use of Stata, version 12 (Stata, College Station, TX, USA). As for one variant to one cancer or non-cancer disease risk, the additive genetic model with at least three independent datasets and on the basis of the minor allele was established in meta-analyses. Moreover, the analyses performed by ethnicity and histological/pathological/clinical subtypes were also recommended if necessary. This study adopted the *I^2^* statistics and Cochran’s *Q* test for investigating the heterogeneity between studies, as well as *P* < 0.1 is the significant level, as recommended (23). The values of *I²* were divided into three parts: ≤ 25%, 25%–50%, and ≥ 50% (mild heterogeneity heterogeneity, moderate heterogeneity, and large heterogeneity, respectively). We conducted sensitive analyses in order to reveal whether the significant ORs were lost by eliminating one individual study, or the first published study, or articles which have been deviated from the Hardy–Weinberg equilibrium (HWE) among controls. We investigated the probability of an excess of significant findings (*P* < 0.1 as the significant level) (24). The Begg’s test and the Egger’s test were used to assess potential publication bias and small-study bias, as well as *P* < 0.1 is the significant level, as recommended (25, 26).

### Epidemiological Credibility of Significant Associations

The epidemiological credibility of significant relationships was evaluated combining the Venice guideline (27) and the false-positive report probability (FPRP) (28) (see **Supplementary Method**).

### Functional Annotation

The underlying functional role of variants on 5p15.33 was evaluated with information from the Encyclopedia of DNA Elements (ENCODE) tool HaploReg (v4.1) (29) and the UCSC Genome browser (http://genome.ucsc.edu/). Furthermore, our work explored genome-wide cis-eQTL data in multiple tissues from the Genotype-Tissue Expression Project (30) and the Multiple Tissue Human Expression Resource Project (31) databases in order to reveal whether these genes might demonstrate the observed findings in these loci.

## Results

### Characteristics of the Included Studies

In our study, available data from 109 papers were extracted in these meta-analyses (**Supplementary Table 2**), thus further evaluating associations between 23 variants in *TERT-CLPTM1L* region and 12 cancers and 1 non-cancer disease under an additive genetic model (**Figure 1**). In addition, the distributions of SNPs (*n*) with cancer and non-cancer disease were as follows: esophageal (*n =* 4), gastric (*n =* 4), glioma (*n =* 2), breast (*n* = 11), hepatocellular (*n* = 1), lung (*n =* 10), pancreatic (*n =* 2), skin (melanoma) (*n =* 1), bladder (*n* = 4), colorectal (*n* = 1), thyroid cancer (*n =* 1), myeloproliferative neoplasms (*n* = 1), and idiopathic pulmonary fibrosis (*n =* 1). Of these, analysis was performed with 139,510 cases and 208,530 controls (the publishing year ranged from 2007 to 2020), and then it was found that 13 SNPs were significantly associated with 11 cancers and 1 non-cancer disease risk (**Table 1** and **Supplementary Table 3**).

**Table 1:** Epidemiological evidence for associations between variants in the TERT and CLPTM1L gene with risk of cancer and non-cancerous diseases in additive model.

Gene	Variant	Alleles^a^	Group^b^	MAF^c^	Number evaluation			Disease risk		Heterogeneity		Venice criteria^d^	FPRP values^e^	Credibility of evidence^f^
					Studies	Cases	Controls	OR (95%CI)	p*-*value	*I^2^* (%)	*P _Q_*			
**Bladder cancer**														
TERT	rs27360986	C/T	Overall	0.3214	5	1,863	3,381	1.193 (1.085–1.313)	< 0.001	47.8	0.104	ABA	0.006	Strong
			Caucasian	0.2534	2	948	1,649	1.132 (0.977–1.311)	< 0.001	0.0	0.886			
			Asian	0.3591	3	915	1,732	1.240 (1.094–1.405)	0.001	70.4	0.033			
TERT	rs2736100	C/A	Overall	0.5597	4	1,638	3,141	0.883 (0.803–0.970)	0.01	0.0	0.507	ABC	0.152	Weak
			Caucasian	0.5122	2	948	1,649	0.924 (0.812–1.051)	0.228	9.0	0.294			
			Asian	0.5912	2	690	1,492	0.837 (0.728–0.961)	0.012	0.0	0.674			
CLPTM1L	rs401681	C/T	Overall	0.3615	4	1,555	2,500	0.852 (0.771–0.941)	0.002	0.0	0.757	AAA	0.029	Strong
			Caucasian	0.6859	1	498	588	0.874 (0.716–1.018)	0.078	NA	NA			
			Asian	0.3376	3	1,057	1,912	0.851 (0.754–0.961)	< 0.001	0.0	0.554			
CLPTM1L	rs402710	C/T	Overall	0.3236	3	1,454	2,179	0.863 (0.772–0.965)	0.01	0.0	0.635	AAA	0.156	Strong
			Caucasian	0.3179	2	948	1,649	0.897 (0.781–1.031)	0.127	0.0	0.810			
			Asian	0.3349	1	506	530	0.804 (0.667–0.969)	0.022	NA	NA			
**Breast cancer**														
TERT	MNS16A	L/S	Caucasian	0.3004	6	4,591	5,159	0.834 (0.714–0.973)	0.021	72.3	0.003	ACC	0.286	Weak
TERT	rs13167280	G/A	Caucasian	0.7312	3	5,057	5,702	0.963 (0.888–1.043)	0.349	0.0	0.681			
TERT	rs2075786	A/G	Caucasian	0.6460	3	5,057	5,702	0.996 (0.942–1.054)	0.902	0.0	0.716			
TERT	rs2735940	A/G	Overall	0.5282	6	5,514	6,640	0.978 (0.913–1.048)	0.534	31.4	0.212			
			Caucasian	0.5324	5	5,446	6,049	1.006 (0.957–1.059)	0.803	83.6	< 0.001			
			Asian	0.4629	1	68	591	2.688 (1.816–3.980)	< 0.001	NA	NA			
TERT	rs2736098	C/T	Caucasian	0.3004	6	4,591	5,159	0.834 (0.714–0.973)	0.021	72.3	0.003	ACC	0.286	Weak
TERT	rs2736100	C/A	Caucasian	0.4677	4	1,262	1,118	1.062 (0.946–1.192)	0.309	0.0	0.976			
TERT	rs2736109	G/A	Overall	0.4663	8	9,457	12,824	0.987 (0.922–1.056)	0.704	59.1	0.017			
			Caucasian	0.4696	7	9,130	12,257	0.995 (0.957–1.035)	0.809	64.8	0.009			
			Asian	0.3343	1	327	567	1.023 (0.841–1.246)	0.817	NA	NA			
TERT	rs2853669	T/C	Caucasian	0.4864	10	8,635	10,358	1.006 (0.942–1.074)	0.870	41.6	0.080			
TERT	rs2853677	G/A	Caucasian	0.4124	3	5,057	5,702	1.036 (0.980–1.095)	0.211	0.0	0.780			
TERT	rs2853690	G/T	Caucasian	0.1496	3	5,057	5,702	0.977 (0.905–1.054)	0.541	0.0	0.847			
TERT	rs7712562	A/C	Caucasian	0.2791	3	5,057	5,702	1.035 (0.927–1.156)	0.540	50.4	0.133			
**Colorectal cancer**														
TERT	rs2736100	C/A	Caucasian	0.4980	10	19,050	21,500	1.070 (1.040–1.102)	< 0.001	0.0	0.700	AAC	< 0.001	Moderate
**Esophageal squamous cell carcinoma**														
TERT	rs2736100	C/A	Asian	0.6055	3	2,098	2,150	0.724 (0.664–0.789)	< 0.001	0.0	0.779	AAA	< 0.001	Strong
TERT	rs2853691	A/G	Asian	0.2486	3	2,098	2,150	1.304 (1.149–1.479)	< 0.001	40.6	0.186	ABA	0.001	Strong
CLPTM1L	rs401681	C/T	Asian	0.3386	3	1,742	1,856	0.867 (0.784–0.958)	0.005	16.2	0.303	AAA	0.088	Strong
CLPTM1L	rs451360	C/A	Asian	0.1258	3	2,098	2,150	0.700 (0.610–0.904)	< 0.001	0.0	0.979	BAA	0.156	Moderate
**Gastric cancer**														
TERT	rs10069690	C/T	Asian	0.1816	4	2,470	2,236	1.317 (1.193–1.454)	< 0.001	28.5	0.241	ABA	< 0.001	Strong
TERT	rs2735940	A/G	Overall	0.3234	3	641	1,528	1.302 (0.689–2.460)	0.416	94.1	< 0.001			
			Caucasian	0.3684	1	104	209	0.695 (0.485–0.996)	0.047	NA	NA			
			Asian	0.3150	2	537	1,319	1.748 (0.894–3.419)	0.103	93.8	< 0.001			
TERT	rs2736100	C/A	Overall	0.6270	4	1,843	2,195	0.751 (0.568–0.993)	0.044	85.1	< 0.001	ACC	0.514	Weak
			Caucasian	0.4880	1	104	209	0.604 (0.429–0.850)	0.004	NA	NA			
			Asian	0.6418	3	1,739	1,986	0.795 (0.580–1.091)	0.155	87.7	< 0.001			
TERT	rs2853676	T/C	Asian	0.7990	4	2,182	2,400	0.675 (0.484–0.942)	0.021	89.5	< 0.001	ACC	0.428	Weak
**Glioma**														
TERT	rs2736100	C/A	Overall	0.5046	8	5,750	8,379	0.746 (0.666–0.835)	< 0.001	77.0	< 0.001	AAA	< 0.001	Strong
			Caucasian	0.4934	6	4,668	7,112	0.796 (0.747–0.847)	< 0.001	25.0	0.246			
			Asian	0.6575	2	1,082	1,267	0.493 (0.255–0.954)	0.036	91.4	0.001			
TERT	rs2853676	T/C	Overall	0.7477	7	5,832	8,143	0.784 (0.743–0.828)	< 0.001	0.0	0.504	AAA	< 0.001	Strong
			Caucasian	0.7263	5	4,423	6,623	0.794 (0.748–0.843)	< 0.001	0.0	0.670			
			Asian	0.8401	2	1,409	1,520	0.737 (0.645–0.843)	< 0.001	49.5	0.159			
**Hepatocellular carcinoma**														
TERT	rs2736098	C/T	Asian	0.3453	3	846	867	1.211 (0.948–1.548)	0.125	65.1	0.057			
**Idiopathic pulmonary fibrosis**														
TERT	rs2736100	C/A	Asian	0.5898	3	397	1,848	1.788 (1.508–2.120)	< 0.001	0.0	0.910	AAA	< 0.001	Strong
**Lung cancer**														
TERT	rs2242652	G/A	Asian	0.1650	3	3,631	4,013	1.168 (1.054–1.294)	0.003	0.0	0.562	AAA	0.053	Strong
TERT	rs2736098	C/T	Overall	0.3199	10	5,350	6,115	1.212 (1.121–1.310)	< 0.001	47.0	0.049	ABA	< 0.001	Strong
			Caucasian	0.2259	1	874	1,061	1.158 (0.989–1.358)	0.069	NA	NA			
			Asian	0.3350	9	4,476	5,054	1.221 (1.118–1.334)	< 0.001	52.3	0.237			
TERT	rs2736098	C/T	NSCLC (ADC)	0.3502	3	1,131	2,203	1.401 (1.261–1.557)^*^	< 0.001	0.0	0.740			
TERT	rs2736098	C/T	NSCLC (SCC)**^1^**	0.3502	3	556	2,203	1.098 (0.954–1.263)^*^	0.193	0.0	0.691			
TERT	rs2736100	C/A	Overall	0.5024	27	33,918	35,844	0.856 (0.788–0.931)	< 0.001	90.9	< 0.001	AAA	0.005	Strong
			Caucasian	0.4927	5	15,861	16,515	0.874 (0.845–0.903)	< 0.001	0.0	0.754			
			Asian	0.5907	21	18,017	19,289	0.847 (0.758–0.948)	0.004	91.9	< 0.001			
			African	0.0972	1	40	40	1.061 (0.541–2.078)	0.864	NA	NA			
TERT	rs2736100	C/A	Overall	0.6028	10	7,730	19,200	0.786 (0.729–0.848)	< 0.001	62.8	0.004			
			Caucasian	0.4801	1	200	553	0.714 (0.566–0.901)	0.005	NA	NA			
			Asian	0.6065	10	7,530	18,647	0.791 (0.731–0.857)	< 0.001	65.7	0.003			
TERT	rs2736100	C/A	NSCLC (ADC)	0.4962	17	8,506	30,944	0.801 (0.694–0.926)**^§^**	0.003	92.7	< 0.001			
			NSCLC (ADC)	0.4801	1	112	553	0.675 (0.503–0.905)**^#^**	0.009	NA	NA			
			NSCLC (ADC)	0.4965	16	8,394	30,391	0.809 (0.698–0.939)^*^	0.005	93.0	< 0.001			
TERT	rs2736100	C/A	NSCLC (SCC)**^1^**	0.5645	4	1,065	2,717	0.920 (0.827–1.025)**^§^**	0.13	0.0	0.458			
			NSCLC (SCC)**^1^**	0.4801	1	48	553	0.741 (0.485–1.132)**^#^**	0.166	NA	NA			
			NSCLC (SCC)**^1^**	0.5861	3	1,017	2,159	0.934 (0.836–1.044)^*^	0.231	0.0	0.467			
TERT	rs2853677	G/A	Asian	0.5881	3	1,123	1,340	0.791 (0.704–0.890)	< 0.001	1.5	0.362	AAA	0.002	Strong
TERT	rs2853677	G/A	NSCLC	0.6796	3	1,085	1,236	0.761 (0.672–0.861)^*^	< 0.001	0.0	0.639			
TERT	rs2853677	G/A	NSCLC (ADC)	0.3255	5	4,443	16,528	1.062 (0.850–1.326)^*^	0.596	94.1	< 0.001			
CLPTM1L	rs31489	C/A	Overall	0.3546	7	13,850	14,390	0.860 (0.813–0.909)	< 0.001	44.7	0.093	AAA	< 0.001	Strong
			Caucasian	0.4068	4	11,361	11,546	0.866 (0.817–0.919)	< 0.001	51.8	< 0.001			
			Asian	0.1496	3	2,489	2,844	0.833 (0.708–0.979)	0.027	51.7	0.126			
CLPTM1L	rs31489	C/A	NSCLC	0.2938	3	1,725	2,343	0.842 (0.603–1.070)	0.159	70.7	0.033			
			NSCLC	0.4098	1	1,154	1,137	0.884 (0.785–0.995)	0.042	NA	NA			
			NSCLC	0.1845	2	571	1206	0.826 (0.505–1.349)	0.444	82.3	0.017			
CLPTM1L	rs401681	C/T	Overall	0.3600	17	11,280	12,390	0.885 (0.840–0.932)	< 0.001	35.5	0.073	AAA	< 0.001	Strong
			Caucasian	0.4284	3	4,306	4,503	0.874 (0.822–0.929)	< 0.001	0.0	0.926			
			Asian	0.3812	14	6,974	7,887	0.891 (0.830–0.957)	0.002	46.7	0.028			
CLPTM1L	rs401681	C/T	NSCLC	0.3527	10	10,109	13,800	0.846 (0.790–0.906)**^§^**	< 0.001	48.0	0.044			
			NSCLC	0.4425	2	4,470	2,575	0.855 (0.797–0.917)**^#^**	< 0.001	0.0	0.649			
			NSCLC	0.3321	8	5,639	11,225	0.853 (0.773–0.940)^*^	0.001	56.7	0.024			
CLPTM1L	rs401681	C/T	NSCLC (ADC)	0.4083	7	2,783	4,780	0.950 (0.886–1.019)**^§^**	0.15	0.0	0.792			
			NSCLC (ADC)	0.4425	2	1,559	2,575	0.939 (0.858–1.027)**^#^**	0.167	57.1	0.127			
			NSCLC (ADC)	0.3666	5	1,224	2,205	0.968 (0.865–1.082)^*^	0.565	0.0	0.960			
CLPTM1L	rs401681	C/T	NSCLC (SCC)**^1^**	0.4083	7	2,283	4,780	0.857 (0.787–0.934)**^§^**	< 0.001	4.4	0.393			
			NSCLC (SCC)**^1^**	0.4425	2	1,819	2,575	0.847 (0.774–0.927)**^#^**	< 0.001	0.0	0.510			
			NSCLC (SCC)**^1^**	0.3666	5	464	2,205	0.876 (0.743–1.033)^*^	0.117	23.5	0.264			
CLPTM1L	rs401681	C/T	NSCLC (SCC)**^2^**	0.3781	4	1,283	3,153	0.908 (0.822–1.003)**^§^**	0.058	0.0	0.712			
			NSCLC (SCC)**^2^**	0.6483	1	1,028	1,438	0.889 (0.793–0.997)**^#^**	0.045	NA	NA			
			NSCLC (SCC)**^2^**	0.3225	3	255	1,715	0.968 (0.792–1.183)^*^	0.747	0.0	0.655			
CLPTM1L	rs402710	C/T	Overall	0.3339	16	20,135	25,250	0.857 (0.832–0.883)	< 0.001	0.0	0.873	AAA	< 0.001	Strong
			Caucasian	0.3515	3	11,190	14,329	0.857 (0.822–0.893)	< 0.001	0.0	0.685			
			Asian	0.3168	11	8,945	10,921	0.858 (0.821–0.896)	< 0.001	0.0	0.747			
CLPTM1L	rs402710	C/T	NSCLC	0.3204	4	5,640	10,521	0.832 (0.785–0.882)	< 0.001	0.0	0.421			
CLPTM1L	rs402710	C/T	NSCLC (ADC)	0.3026	2	2,099	3,329	0.868 (0.796–0.947)	0.002	0.0	0.587			
TERT	rs4246742	T/A	Asian	0.6252	3	3,305	3,720	1.133 (0.875–1.467)	0.343	82.0	0.004			
CLPTM1L	rs465498	A/G	Asian	0.1701	4	8,948	9,805	0.765 (0.723–0.810)	< 0.001	0.0	0.880	AAA	< 0.001	Strong
TERT/CLPTM1L	rs4975616	G/A	Overall	0.6387	7	11,300	8,873	1.159 (1.108–1.212)	< 0.001	32.7	0.146	AAA	< 0.001	Strong
			Caucasian	0.5896	4	9,553	7,213	1.159 (1.105–1.216)	< 0.001	56.1	0.044			
			Asian	0.8517	3	1,747	1,660	1.155 (1.006–1.326)	0.041	0.0	0.577			
TERT/CLPTM1L	rs4975616	G/A	NSCLC	0.6029	3	1,554	1,890	1.234 (0.979–1.556)^§^	0.075	66.1	0.052			
			NSCLC	0.5731	2	1,354	1,690	1.279 (0.929–1.761)**^#^**	0.132	82.8	0.016			
			NSCLC	0.8550	1	200	200	1.110 (0.743–1.659)^*^	0.609	NA	NA			
**Myeloproliferative neoplasms**														
TERT	rs2736100	C/A	Overall	0.5846	7	2,436	19,204	0.586 (0.538–0.637)	< 0.001	0.0	0.848	AAA	< 0.001	Strong
			Caucasian	0.4925	4	1,956	1,791	0.589 (0.532–0.654)	< 0.001	0.0	0.552			
			Asian	0.5940	3	480	17,413	0.578 (0.498–0.670)	< 0.001	0.0	0.764			
TERT	rs2736100	C/A	ET	0.5909	4	792	18,145	0.589 (0.522–0.665)^§^	< 0.001	0.0	0.707			
			ET	0.5228	2	552	833	0.561 (0.481–0.655)**^#^**	< 0.001	0.0	0.536			
			ET	0.5942	2	240	17,312	0.637 (0.525–0.774)^*^	< 0.001	0.0	0.970			
TERT	rs2736100	C/A	PV	0.5909	4	499	18,145	0.521 (0.449–0.604)^§^	< 0.001	46.7	0.131			
			PV	0.5228	2	393	833	0.562 (0.473–0.669)**^#^**	< 0.001	0.0	0.745			
			PV	0.5942	2	106	17,312	0.422 (0.318–0.562)^*^	< 0.001	64.7	0.092			
TERT	rs2736100	C/A	PMF	0.5909	4	201	18,145	0.575 (0.463–0.713)^§^	< 0.001	0.0	0.472			
			PMF	0.5228	2	168	833	0.538 (0.423–0.686)**^#^**	< 0.001	2.3	0.312			
			PMF	0.5942	2	33	17,312	0.758 (0.464–1.237)^*^	0.267	0.0	0.908			
**Pancreatic cancer**														
CLPTM1L	rs401681	C/T	Caucasian	0.4398	3	2,591	5,383	1.173 (1.097–1.255)	< 0.001	0.0	0.974	AAA	< 0.001	Strong
TERT/CLPTM1L	rs4635969	G/A	Caucasian	0.2057	3	2,591	5,383	1.026 (0.943–1.117)	0.547	36.3	0.208			
**Skin cancer (melanoma)**														
CLPTM1L	rs401681	C/T	Caucasian	0.4415	3	1,188	1,603	1.285 (1.120–1.414)	< 0.001	23.5	0.270	AAA	0.002	Strong
**Thyroid cancer**														
TERT	rs2736100	C/A	Asian	0.5969	4	2,752	2,752	0.762 (0.657–0.884)	< 0.001	72.2	0.013	ACC	0.007	Moderate

OR, odds ratio; A, adenine; T, thymine; G, guanine; C, cytosine; NSCLC, non–small cell lung cancer; ADC, adenocarcinoma; ^1^SCC, squamous cell carcinoma. ^2^SCC, small cell cancer; ET, essential thrombocythemia; PV, polycythemia vera; PMF, primary myelofibrosis; NA, not applicable.

^*^The association was performed in Asians.

**^#^** The association was performed in Caucasians.

^§^The association was performed in overall populations.

a Major alleles (reference)/minor alleles.

b Group by ethnicity or subtype.

c Frequency of minor allele in controls.

d Strength of epidemiological evidence based on the Venice criteria.

e FPRP values at prior probability of 0.05 at power OR of 1.5, and the FPRP level of noteworthiness is 0.20.

f Degree of epidemiological credibility based on the combination of results from Venice guidelines and FPRP tests.

### Associations Between TERT-CLPTM1L Variants and Risk of Cancer and Non-Cancer Diseases

We conducted meta-analyses to assess relationships of 23 variants in *TERT-CLPTM1L* region with 12 cancers and 1 non-cancer disease under an additive genetic model. Then, it was seen that 13 SNPs (rs10069690, rs2242652, rs2736098, rs2736100, rs2853676, rs2853677, rs2853691, rs31489, rs401681, rs402710, rs451360, rs465498, and rs4975616) had significantly associated with risk of 11 cancers (bladder, breast, colorectal, esophageal, gastric, glioma, lung, pancreatic, skin, thyroid, and myeloproliferative neoplasms) and 1 non-cancer disease (idiopathic pulmonary fibrosis) (**Table 1**). It is worth noting that the histological/pathological types of esophageal carcinoma and skin cancer were squamous cell carcinoma and melanoma, respectively. Apart from that, significant relationships with susceptibility to bladder cancer could be found for rs2736098 (OR = 1.193), rs2736100 (OR = 0.883), rs401681 (OR = 0.852), and rs402710 (OR = 0.863). Moreover, these associations were further assessed by ethnicity, demonstrating that the four SNPs mentioned above had significant association with bladder cancer predisposition in Asians (rs2736098: OR = 1.240; rs2736100: OR = 0.837; rs401681: OR = 0.851; rs402710: OR = 0.804), rather than the Caucasian population. In addition, the significant relationship with breast cancer predisposition was only presented for rs2736098 (OR = 0.834), whereas rs2736100 could increase colorectal cancer predisposition (OR = 1.070).

Apart from colorectal carcinoma, the A allele of rs2736100 possessed a decreased predisposition of esophageal squamous cell carcinoma (ESCC) among Asian populations (OR = 0.724). Moreover, one SNP (rs2853691) had an enhanced predisposition of ESCC (OR = 1.304) and another two SNPs (rs401681 and rs451360) were featured by the reduced predisposition of ESCC, in Asians (OR = 0.867 and OR = 0.700).

Significant relationships with gastric carcinoma predisposition were exclusively found for rs10069690 (OR = 1.317) and rs2853676 (OR = 0.675) in Asians. Besides, SNP rs2736100 was statistically associated with gastric carcinoma (OR = 0.751). Interestingly, rs2736100 remarkably associates with gastric cancer predisposition in Caucasians (OR = 0.604) but not in Asians. Moreover, two SNPs could reduce the predisposition of glioma (rs2736100: OR = 0.746; rs2853676: OR = 0.784). Noticeably, it was also uncovered that these two SNPs had a reduced susceptibility to glioma both in Asian populations and Caucasian populations.

For lung cancer, it was found that nine SNPs were significantly related to lung cancer predisposition, which also remains true in the subgroup analyses by ethnicity and pathological subtypes. Specifically, three SNPs exclusively appeared in Asians and had significant relationships with lung cancer predisposition (rs2242652: OR = 1.168; rs2853677: OR = 0.791; rs465498: OR = 0.765, respectively). Besides, additional findings from subgroup analyses by pathological subtypes for rs2853677 indicated that this SNP could decrease the predisposition of non–small cell lung cancer (NSCLC) (OR = 0.761). Interestingly, it was found that rs2853677 had no association with NSCLC (adenocarcinoma) risk. In addition, there were noticeable relationships between six SNPs and lung cancer predisposition among different races/pathological subtypes. Among them, rs2736098 had an elevated predisposition of lung cancer (OR = 1.212), which was shown in Asians (OR = 1.221), but not in Caucasians. Besides that, rs2736098 was distinctly associated with predisposition of NSCLC (adenocarcinoma) (OR = 1.401), rather than NSCLC (squamous cell carcinoma), whereas SNP rs2736100 had a close relationship with lung cancer predisposition (OR = 0.856), which appeared in Caucasians (OR = 0.874) and Asians (OR = 0.847) instead of Africans. Then, subgroup analyses were performed by pathological type/race, and it was uncovered that rs2736100 had a decreased risk of NSCLC (OR = 0.786) both in Caucasians (OR = 0.714) and Asians (OR = 0.791). Surprisingly, rs2736100 had a decreased risk of NSCLC (adenocarcinoma) (OR = 0.801), rather than NSCLC (squamous cell carcinoma), whereas SNP rs31489 was closely connected with lung cancer predisposition (OR = 0.860) both in Caucasians (OR = 0.866) and Asians (OR = 0.833). Noticeably, subgroup analyses by pathology type presented that rs31489 was not related to NSCLC predisposition (OR = 0.842). Additionally, SNP rs401681 was featured by the decreased lung cancer incidence (OR = 0.885) both in Caucasians (OR = 0.874) and Asians (OR = 0.891). Furthermore, the analyses also showed that rs401681 had a decreased risk of NSCLC (OR = 0.846), but not associated with small cell carcinoma (OR = 0.908). For NSCLC, it was also known that SNP rs401681 featured a reduced NSCLC (squamous cell carcinoma) incidence (OR = 0.857) but had no relationship with NSCLC (adenocarcinoma) (OR = 0.950). Other than that, SNP rs402710 could decrease predisposition of lung cancer (OR = 0.857) both in Caucasians (OR = 0.857) and Asians (OR = 0.858). Furthermore, from subgroup analyses by pathology type, it was seen that rs401681 could reduce NSCLC predisposition (OR = 0.832), especially in lung adenocarcinoma (OR = 0.868). SNP rs4975616 faced an enhanced risk of lung cancer (OR = 1.159) both in Caucasians (OR = 1.159) and Asians (OR = 1.155), while there was no relationship between rs4975616 and NSCLC predisposition (OR = 1.234).

For myeloproliferative neoplasms, SNP rs2736100 could decrease the risk of myeloproliferative neoplasms (OR = 0.586) both in Caucasians (OR = 0.589) and in Asians (OR = 0.578). Subgroup analyses by clinical subtypes indicated that rs2736100 could reduce the risk of essential thrombocythemia (OR = 0.589), polycythemia vera (OR = 0.521), and primary myelofibrosis (OR = 0.575).

Besides myeloproliferative neoplasms, SNP rs2736100 could reduce thyroid carcinoma predisposition in Asians (OR = 0.762). Moreover, SNP rs401681 could increase risk of pancreatic cancer (OR = 1.173) and skin cancer (melanoma) (OR = 1.285) in Caucasians.

In terms of non-cancer disease, it was found that rs2736100 had an increased risk of idiopathic pulmonary fibrosis in Asian populations (OR = 1.788).

Furthermore, 13 SNPs (*TERT* MNS16A, rs13167280, rs2075786, rs2735940, rs2736100, rs2736109, rs2853669, rs2853677, rs2853690, rs7712562, rs2735940, rs2736098, rs4246742, and rs4635969) were not related to risk of five types of cancer (breast, gastric, hepatocellular, lung, and pancreatic cancer) (**Supplementary Results**). Of these, eight SNPs (rs13167280, rs2075786, rs2735940, rs2736100, rs2736109, rs2853669, rs2853677, rs2853690, and rs7712562) had no association with breast cancer with at least 10,000 individuals (**Table 2**). Beyond that, the statistical power was also calculated so as to confirm whether the large-scale sample size confirming these associations is required in the future (**Supplementary Table 4**).

**Table 2:** Summary of functional annotations for 12 SNPs in eight cancers and one neoplastic disease (strong epidemiological credibility).

Variant	Gene	Position^a^	Annotation	Promoter histone marks^b^	Enhancer histone marks^c^	DNAse^d^	Proteins bound^e^	Motifs changed^f^
rs10069690	TERT	1279790	Intronic	4 tissues	4 tissues			BDP1, TBX5
rs2242652	TERT	1280028	Intronic	4 tissues	4 tissues			9 altered motifs
rs2736098	TERT	1294086	Synonymous	10 tissues	16 tissues	BLD		9 altered motifs
rs2736100	TERT	1286516	Intronic	ESDR, ESC	BLD			Foxa
rs2853676	TERT	1288547	Intronic	ESDR, PANC, SPLN	BLD			Pax-5
rs2853677	TERT	1287194	Intronic	ESDR, ESC	BLD			
rs2853691	TERT	1252950	Intronic	4 tissues	4 tissues			6 altered motifs
rs31489	CLPTM1L	1342714	Intronic	13 tissues	17 tissues			DMRT2, Mef2
rs401681	CLPTM1L	1322087	Intronic	5 tissues	6 tissues			Egr-1, HNF4
rs402710	CLPTM1L	13947292	Intronic	4 tissues	7 tissues			5 altered motifs
rs465498	CLPTM1L	1325803	Intronic	9 tissues	13 tissues			RXRA, Rad21
rs4975616	TERT/CLPTM1L	1315660	2.2kb 3’ of CLPTM1L	11 tissues	18 tissues	6 tissues		NF-I

a The chromosome position is based on NCBI Build 37.

b Histone modification of H3K4me1 and H3K27ac (tissue types: if >3, only the number is included).

c Histone modification of H3K4me3 and H3K9ac (tissue types: if >3, only the number is included).

d Levels of DNase I hypersensitivity (tissue types: if >3, only the number is included).

e Alteration in transcription factor binding (disruptions: if >3, only the number is included).

f Alteration in regulatory motif (disruptions: if >3, only the number is included).

### Heterogeneity, Bias, and Sensitivity Analysis

The assessment of heterogeneity was performed for 29 significant correlations with 13 variants and 12 cancers and 1 non-cancer disease (**Table 1**). Of them, mild heterogeneity could be assigned to 16 (55%) associations, moderate heterogeneity fell into 7 (24%) associations, and high heterogeneity was found in 6 (21%) associations. There existed little evidence of publication bias (*p* > 0.10), except for rs2853676 with risk of glioma. Furthermore, findings from sensitivity analyses displayed that removal of some key factors did not alter the summary ORs, except for rs2736100 in bladder and colorectal cancer (low OR), rs2736100 in gastric cancer (HWE), and rs2736100 in thyroid cancer (excess of significant findings).

### Cumulative Evidence of Association

Epidemiological credibility of totally 29 significant relationships was assessed using the Venice guideline. Specifically, there were 28 grades A in the amount of evidence, 21 grades A in the replication of association, and 23 grades A in the protection from bias, respectively; there were 1, 4, and 0 grades B in these three criteria, respectively; and there were 0, 4, and 6 grades C in these three criteria, respectively (**Table 1**). Therefore, strong, moderate, and weak evidence of a significant relationship with susceptibility to cancer and non-cancer disease could be found for 18, 5, and 6 associations, respectively. Subsequently, the probability for a true correlation between the 29 significant correlations was evaluated on the basis of FPRP values. Briefly, a FPRP level < 0.05 was found for 11 variants with 10 cancers and 1 non-cancer disease, whereas FPRP 0.05 to 0.2 for five variants with susceptibility to three cancers, and FPRP > 0.2 for three SNPs with risk of two cancers. Finally, strong evidence was assigned to 12 SNPs (*TERT*: rs10069690, rs2242652, rs2736098, rs2736100, rs2853676, rs2853677, and rs2853691; *CLPTM1L*: rs31489, rs401681, rs402710, and rs465498; *TERT/CLPTM1L*: rs4975616) with eight cancers and one non-cancer disease, which is presented in detail below: rs2736098, rs401681, and rs402710 in bladder cancer; rs2736100, rs2853691, and rs401681 in esophageal cancer; rs10069690 in gastric cancer; rs2736100 and rs2853676 in glioma; rs2242652, rs2736098, rs2736100, rs2853677, rs31489, rs401681, rs402710, rs465498, and rs4975616 in lung cancer; rs2736100 in idiopathic pulmonary fibrosis and myeloproliferative neoplasms; and rs401681 in pancreatic and skin cancer, whereas moderate evidence belonged to two SNPs with risk of three cancers (rs2736100 in colorectal and thyroid cancer and rs451360 in esophageal cancer), and the weak one was to three SNPs with risk of three cancers (rs2736100 in bladder cancer, rs2736098 in breast cancer, and rs2736100 and rs2853676 in gastric cancer).

### Functional Annotation

**Figure 2:**
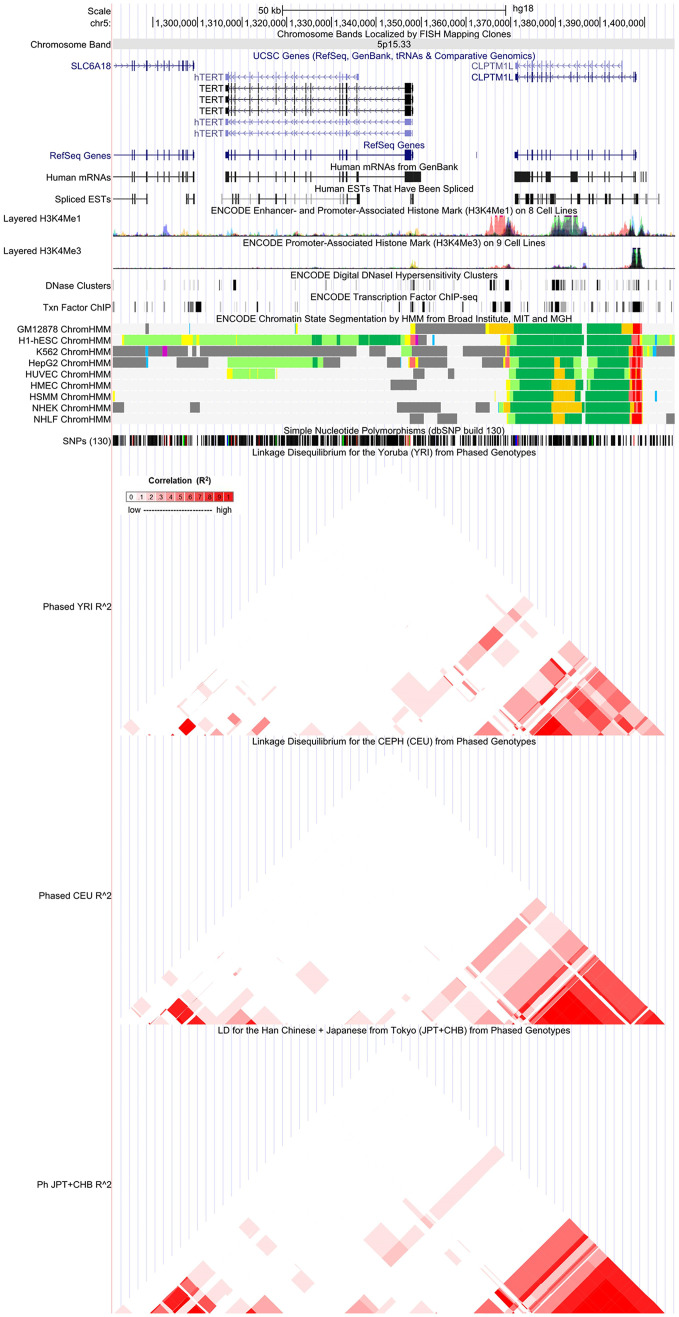
Evidence from the Encyclopedia of DNA Elements (ENCODE) data for regulatory function of variants in 5p15.33 using the UCSC Genome Browser. The plot represents *5p15.33* within a 50-kb window centered on *TERT-CLPTM1L* gene region. Tracks (from top to bottom) in each of the plots are genome base position, chromosome bands, UCSC genes, human messenger RNAs from GenBank, human-expressed sequence tag (ESTs) that have been spliced, ENCODE enhancer and promoter-associated histone mark (H3K4Me1) on 8 Cell Lines, ENCODE promoter-associated histone mark (H3K4Me3) on 9 cell lines, ENCODE digital DNaseI hypersensitivity clusters, ENCODE transcription factor ChIP-seq, ENCODE chromatin state segmentation by hidden Markov model (HMM) from Broad Institute (bright red, active promoter; light red, weak promoter; purple, inactive/poised promoter; orange, strong enhancer; yellow, weak/poised enhancer; blue, insulator; dark green, transcriptional transition/elongation; light green, weak transcribed; gray, polycomb-repressed; light gray, heterochromatin/low signal/repetitive/copy number variation), single-nucleotide polymorphisms (dbSNP build 130), linkage disequilibrium for the Yoruba (YRI) from phased genotypes, linkage disequilibrium for the CEPH (CEU) from phased genotypes and LD for the Han Chinese + Japanese from Tokyo (JPT+CHB) from Phased Genotypes. The scale bar for the LD plot could be found in the data source (ldlink.nci.nih.gov/?tab=ldpair.).

The potential function roles for strong associations (12 SNPs associated with eight cancers and one non-cancer disease) were investigated with the use of the ENCODE tool HaploReg v4.1 (**Table 2**). In terms of functional annotations, 10 variants were mapped to intronic regions and *TERT* rs2736098 was mapped to synonymous regions. The total 12 SNPs might locate in a region having strong promoter and enhancer activity, DNase I hypersensitivity site, and alteration in regulatory motifs (**Table 2**). The linkage disequilibrium (LD) plots explained that the regions represented by significant SNPs had distinct genetic structures among European, Asian, and African ancestries (**Figure 2** **)**. Besides, the Genotype-Tissue Expression Project revealed that rs2736100, rs2853676, rs2853676, rs31489, rs401681, rs402710, rs465498, and rs4975616 are eQTLs for *TERT* and *CLPTM1L*. Additionally, rs2736100, rs2853676, rs2853677, and rs4975616 are associated with an increase in *TERT* and *CLPTM1L* gene expression, whereas rs31489, rs401681, rs402710, and rs465498 are relevant to a decrease in *CLPTM1L* gene expression in skin tissues; different from that, rs31489, rs401681, rs402710, and rs465498 relate to a decrease, but rs4975616 to an increase in *CLPTM1L* gene expression in esophagus tissues; apart from that, rs31489, rs401681, and rs465498 are connected with an increase, but rs4975616 with a decrease in the *CLPTM1L* gene in stomach tissues (**Supplementary Table 5**).

## Discussion

Admittedly, the current large-scale research synopsis and meta-analysis comprehensively summarize and update the correlations between variants in *TERT-CLPTM1L* genes and cancer and non-cancer disease predisposition, which provides precise results for the variants and offers more SNPs and diseases that were never assessed before. To be specific, in this paper, meta-analyses were performed by employing available data from 109 papers with 139,510 cases and 208,530 controls, thus evaluating associations of 23 SNPs with risk of 12 cancers and 1 non-cancer disease; then, it was revealed that, among them, 13 SNPs had significant association with 11 cancers and 1 non-cancer disease predisposition. Besides, the Venice guidelines and FPRP tests were taken for the first time to assess these significant correlations. At last, 12 variants were rated as being strong for cumulative evidence with eight cancers and one non-cancer disease predisposition (22 significant associations: rs2736098, rs401681, and rs402710 in bladder cancer; rs2736100, rs2853691, and rs401681 in esophageal cancer; rs10069690 in gastric cancer; rs2736100 and rs2853676 in glioma; rs2242652, rs2736098, rs2736100, rs2853677, rs31489, rs401681, rs402710, rs465498, and rs4975616 in lung cancer; rs401681 in pancreatic and skin cancer; and rs2736100 in myeloproliferative neoplasms and idiopathic pulmonary fibrosis). Moreover, the study here tended to construct functional annotations for these 12 SNPs with strong evidence using information from the ENCODE Project and other public databases and subsequently revealed that these variants might fall in several putative regulatory regions. Briefly, our research offers comprehensive epidemiological evidence that common variants in the *TERT-CLPTM1L* region show association with predisposition of glioma, myeloproliferative neoplasms, idiopathic pulmonary fibrosis, esophageal cancer, gastric cancer, skin cancer, bladder cancer, lung cancer, and pancreatic cancer.

The *TERT* gene (Gene ID: 7015), encoding the enzyme of *TERT*, plays crucial roles in maintaining the telomere length (32, 33). In the previous research, it was pointed out that telomere length had linked with glioma, ovarian cancer, lung cancer, and melanoma predisposition, rather than breast and prostate cancer (34, 35). Besides, a recent meta-analysis on the GWAS revealed that the variants in *TERT-CLPTM1L* genes may affect cancer risk through a variety of different biological pathways, and telomere length is the only one of the related mechanisms (36), whereas in our study, strong evidence was given to seven SNPs (rs10069690, rs2242652, rs2736098, rs2736100, rs2853676, rs2853677, and rs2853691) in *TERT*. Four SNPs (rs2853677, rs2242652, rs2736098, and rs2736100) were related to the predisposition of lung cancer. The phase 3 of the 1000 Genomes Project (37) (**Supplementary Table 6**) presented that rs2853677 is in moderate LD with rs2736098 (in East Asians: *r* ^2^ = 0.3070) and rs2736100 (in East Asians: *r* ^2^ = 0.5789, in Europeans: *r* ^2^ = 0.4352), is in weak LD with rs2736098 (in Europeans: *r* ^2^ = 0.1504), and is uncorrelated with rs2736098 and rs2736100 in Africans (*r* ^2^ < 0.05). Moreover, rs2242652 is in moderate LD with rs2736098 in Europeans (*r* ^2^ = 0.1504) while is uncorrelated both in East Asians and Africans (*r* ^2^ < 0.05). According to the above findings, the functional mechanisms of the four variants related to lung cancer risk might be different in various ethnic groups, partially accounting for why some variants are demonstrated to be related to a cancer site in one ethnic group but not in others. In addition, two SNPs (rs2736100 and rs2853676) were associated with glioma risk; however, SNP rs2736100 is in weak LD with rs2853676 in East Asians (*r* ^2^ = 0.2057), Europeans (*r* ^2^ = 0.2475), and Africans (*r* ^2^ = 0.1062). To sum up, these data indicated that there might exist different causal variants and functional mechanisms involved associated with variants in the *TERT* gene with predisposition of glioma. Apart from that, SNP rs2736100 and SNP rs2853691 are intron variants of the *TERT* gene, and they are linked with predisposition of esophageal cancer in our study; while in our study, it was found that rs2736100 is unrelated to rs2853691 in Europeans, East Asians, and Africans (*r* ^2^ < 0.05 for all tests), revealing that there might exist various functional mechanisms for relationships of *TERT* variants with predisposition of esophageal cancer. Current evidence presents that the rs10069690 T allele can trigger the development of the coproduction of full-length *TERT* and an alternatively spliced by creating splice donor site in intron 4 of *TERT*, which may increase gastric cancer risk by reducing telomerase activity and telomere shortening (38). Moreover, rs2242652 allele (G > A) could influence telomere length, which could increase predisposition for lung cancer in Asians (39), whereas SNP rs2736098 could cause the overexpression of *TERT* and increase telomerase activity, which regulated bladder and lung cancer development by modulating unlimited cell division, and carcinogenesis, and interacted with the activation of the glycolytic pathway (40). Previous study showed that the intron 2 segment including the rs2736100 flanking sequence proved promoter activities in ESCC cell lines and uncovered an elevated association with ESCC predisposition in carriers of rs2736100 G allele (41), which demonstrated the oncogene inherent characteristics of *TERT* in ESCC. Here, it should be noted that the T allele of rs2736100 could lead to telomere length shortening and then increase lung cancer and non-cancerous disease predisposition (like chronic obstructive pulmonary disease and idiopathic pulmonary fibrosis) (42, 43).

The *CLPTM1L* gene (Gene ID: 81037), encoding the cleft lip and palate–associated transmembrane 1–like protein, could arouse cell apoptosis and cytokinesis (44, 45). In our study, strong evidence was assigned to four SNPs (rs31489, rs401681, rs402710, and rs465498) in *CLPTM1L* and one SNP rs4975616 in *TERT-CLPTM1L*. Of the two variants associated with bladder cancer, rs401681 is in strong LD with rs402710 in East Asians (*r* ^2^ = 0.9371) while has moderate LD in Europeans (*r* ^2^ = 0.6624) and Africans (*r* ^2^ = 0.5961). Moreover, four SNPs (rs31489, rs401681, rs402710, and rs465498) were related to lung cancer, and, in our study, it was found that rs31489 is in strong LD with rs401681 in Europeans (*r* ^2^ = 0.8145) while is in moderate LD in East Asians (*r* ^2^ = 0.4785) and Africans (*r* ^2^ = 0.6131); rs31489 is in strong LD with rs402710 in Africans (*r* ^2^ = 0.8376) while is in moderate LD in East Asians (*r* ^2^ = 0.4634) and Europeans (*r* ^2^ = 0.6624); rs31489 is in strong LD with rs465498 both in East Asians and Europeans (*r* ^2^ > 0.809) while is in moderate LD in Africans (*r* ^2^ = 0.6572). Moreover, it was revealed that rs401681 is in strong LD with rs402710 in East Asians (*r* ^2^ = 0.9371) but shows moderate LD in Europeans (*r* ^2^ = 0.6624) and Africans (*r* ^2^ = 0.5961); rs401681 is in strong LD with rs465498 both in Europeans and Africans (*r* ^2^ > 0.809) while is in moderate LD in East Asians (*r* ^2^ = 0.4839). Based on the obtained results, the functional mechanisms of the four variants associated with risk of bladder and lung cancer may be different in different ethnic groups and partly account for why some variants are discovered to be related to a cancer site in one ethnic group but not in others. Furthermore, current evidence demonstrates that rs31489, a variant in which C is changed to A in *CLPTM1L* gene, could influence the telomere length that could decrease the risk of nonsmokers’ lung carcinoma (rather than in smokers), because smoking can counteract the protective role of A allele, shorten telomere length, and enhance telomerase activity (46). Interestingly, rs401681 could affect transcription regulation, result in the over-expression of the *CLPTM1L* gene, and increase risk of lung and skin carcinoma (47). Moreover, *CLPTM1L* rs402710 may affect lung tissue tumorigenesis *in vitro* by blocking DNA damage–induced apoptosis *via* enhanced accumulation of *Bcl-xL*, an antiapoptotic *Bcl2* family member (48). Beyond that, SNP rs402710 could maintain the telomere length which could decrease the risk of nonsmokers’ lung cancer since the protective role of rs2736100 was counteracted in patients with bladder cancer who currently were smokers (49). In addition, seven variants in the *TERT* gene are uncorrelated or had weak LD with the four variants in the *CLPTM1L* gene in European, Asian, and African populations. According to the results, there exist different causal variants and functional mechanisms in relationships of variants in the *TERT-CLPTM1L* regions with idiopathic pulmonary fibrosis, myeloproliferative neoplasms, and glioma, as well as esophageal, gastric, bladder, lung, pancreatic, and skin cancer predisposition.

In addition, 13 SNPs had no association with five cancer risk in additive model. Of these, eight SNPs (rs13167280, rs2075786, rs2735940, rs2736100, rs2736109, rs2853669, rs2853677, rs2853690, and rs7712562) had no association with breast cancer with at least 5,000 case and 5,000 controls in additive model, which had approximately 98% statistical power to detect an OR of 1.15 for a variant with MAF 0.20 and 86% power with MAF 0.10 (type 1 error 0.05). Therefore, further research with a smaller sample size on these eight SNPs for breast cancer in Caucasians will not be helpful in evaluating effects of those SNPs (**Supplementary Result**, **Table 3** and **Supplementary Table 4**).

**Table 3:** Variants in *TERT-CLPTM1L* showing no relation to breast cancer risk in meta-analyses with at least 5000 cases and 5000 controls in additive model.

Gene	Variant	Allelesa	Cancer Type	Ethnicity	MAFb	Number evaluation		Meta-analysis risk		Heterogeneity	
						Studies	Sample size (case/control)	OR (95%CI)	*P*_value_	I^2^(%)	P _(Q)_
TERT	rs13167280	G/A	Breast cancer	Caucasian	0.7312	3	10759 (5057/5702)	0.963 (0.888-1.043)	0.349	0.0	0.681
TERT	rs2075786	A/G	Breast cancer	Caucasian	0.6460	3	10759 (5057/5702)	0.996 (0.942-1.054)	0.902	0.0	0.716
TERT	rs2735940	A/G	Breast cancer	Overall	0.5282	6	12154 (5514/6640)	0.978 (0.913-1.048)	0.534	31.4	0.212
TERT	rs2735940	A/G	Breast cancer	Caucasian	0.5324	5	11495 (5446/6049)	1.006 (0.957-1.059)	0.803	83.6	< 0.001
TERT	rs2736109	G/A	Breast cancer	Overall	0.4663	8	22281 (9457/12824)	0.987 (0.922-1.056)	0.704	59.1	0.017
TERT	rs2736109	G/A	Breast cancer	Caucasian	0.4696	7	21387 (9130/12257)	0.995 (0.957-1.035)	0.809	64.8	0.009
TERT	rs2853669	T/C	Breast cancer	Caucasian	0.4864	10	18993 (8635/10358)	1.006 (0.942-1.074)	0.870	41.6	0.080
TERT	rs2853677	G/A	Breast cancer	Caucasian	0.4124	3	10759 (5057/5702)	1.036 (0.980-1.095)	0.211	0.0	0.780
TERT	rs2853690	G/T	Breast cancer	Caucasian	0.1496	3	10759 (5057/5702)	0.977 (0.905-1.054)	0.541	0.0	0.847
TERT	rs7712562	A/C	Breast cancer	Caucasian	0.2791	3	10759 (5057/5702)	1.035 (0.927-1.156)	0.540	50.4	0.133

OR, odds ratio; A, adenine; C, cytosine; G, guanine; T, thymine.

a Major alleles (reference)/minor alleles.

b Frequency of minor allele in controls.

In fact, our study has several limitations: (i) although a comprehensive research on databases was conducted, some publications may have been missed, as well as the papers with insufficient data such as the genotype amount, which might result in incomplete assessment of other malignancies (lymphoma, gallbladder cancer, cervical cancer, etc.) and non-cancer disease (chronic hepatitis B, Alzheimer’s disease, diabetes mellitus, etc.); (ii) the potential publication bias might be found due to the usage of the search approach (only search for English papers); (iii) as the subgroup analyses according to ethnicity and partial pathological/clinical subtypes were only performed on lung cancer, idiopathic pulmonary fibrosis and myeloproliferative neoplasms, further analyses based on subgroups such as pathological type, gene-gene or gene-environment associations and interactions, could be required to confirm or refute the correlations with risk of cancers and non-cancer disease; (iv) potential bias for variants with cancers and non-cancer risk could be evaluated by the Venice criteria; however, the unreasonable data, like errors in genotype, could not be evaluated; and (v) meta-analyses were conducted on the basis of the minor allele of a variant; therefore, a protective association for some variants might be found because of the inherent factors in meta-analysis (for example, rs2736100 and rs2853676 could reduce the risk of glioma, myeloproliferative neoplasms, and lung cancer and rs401681 could decrease risk of bladder cancer). Given that, all genetic associations in the current work should be further confirmed and clarified by doing the molecular biology experiments.

To conclude, in our study, it was identified that 12 variants in the *TERT-CLPTM1L* genes were rated as revealing strong evidence for a significant correlation with eight cancers and one non-cancer disease risk. Moreover, our study offers foundation for further demonstrating that the variants in the *TERT-CLPTM1L* genes are related to the risk of idiopathic pulmonary fibrosis, myeloproliferative neoplasms, glioma, esophageal cancer, gastric cancer, bladder cancer, lung cancer, pancreatic cancer, and skin cancer. Apart from that, the crucial roles of the *TERT-CLPTM1L* region in the etiology of human diseases were highlighted.

## Data Availability Statement

The original contributions presented in the study are included in the article/**Supplementary Material**. Further inquiries can be directed to the corresponding author.

## Author Contributions

JT, YW, and GC designed this work. JT and YW integrated and analyzed the data. JT, YW, and GC wrote this manuscript. JT, YW, YD, JC, YMW, SC, and GC finished the related Tables and Figures. JT, YW, and GC edited and revised the manuscript. All authors approved this manuscript.

## Funding

This work was supported by the Sichuan Science and Technology Program (grant No. 2020YFS0252).

## Conflict of Interest

The authors declare that the research was conducted in the absence of any commercial or financial relationships that could be construed as a potential conflict of interest.

## Publisher’s Note

All claims expressed in this article are solely those of the authors and do not necessarily represent those of their affiliated organizations, or those of the publisher, the editors and the reviewers. Any product that may be evaluated in this article, or claim that may be made by its manufacturer, is not guaranteed or endorsed by the publisher.
