# Supplementary material for: Cumulative Evidence for Relationships Between Multiple Variants in the TERT and CLPTM1L Region and Risk of Cancer and Non-Cancer Disease
Source: Front Oncol. 2022 Jun 30;12:946039. doi: 10.3389/fonc.2022.946039 (PMC9279858; doi:10.3389/fonc.2022.946039)
Supplement: Supplementary file 1 [file DataSheet_1.pdf]

Supplementary Method:

Method for Venice Criteria

We applied the Venice Criteria to evaluate the epidemiological credibility of significant associations identified by meta-analysis [(1)]. Specifically, the level of credibility was defined as strong, moderate or weak on the basis of three criteria including amount of evidence, replication and protection from bias, with each criterion assigned with grades of A, B or C. The criterion of amount of evidence was graded by the total number of the tested alleles or genotypes in cases and controls: A for >1000, B for 100–1000 and C for <100. The criterion of replication was graded by the I^2 value: A for $I^2 < 25\%$, B for I^2 between 25% and 50% and C for $I^2 > 50\%$. It might be reasonable to assign grade A to associations with moderate or high heterogeneity on the criterion of replication if they had extensive replication record such as GWAS and GWAS meta-analysis from large collaborative efforts [(2)]. The criterion of protection from bias was graded by a series of sensitivity analyses and bias tests: (i) grade A would be assigned if there was no evident bias or the bias could not explain the existence of association; (ii) grade B would be assigned if there was moderate bias and (iii) grade C would be assigned if bias was obvious or the bias could affect the presence of association. We also took the magnitude of an association into consideration when assessing the protection from bias. If the summary OR was <1.15 (or >0.87 in a protection effect), grade C would be assigned on this criterion except that this association had been replicated extensively by large collaborative studies including GWAS or GWAS meta-analysis, showing no evidence of bias [(2)]. Consequently, the level of credibility of epidemiological evidence was considered to be strong if grades of A were assigned to all three criteria, moderate if the grades were A or B, and weak if there was grade C for any category [(3)].

The checklist of Venice Criteria was presented as follow,

Amount of evidence

A: Large-scale evidence — minor genetic group (alleles or genotypes) in cases and controls > 1,000.

B: Moderate amount of evidence — minor genetic group in cases and controls between 100 and 1,000.

C: Little evidence — minor genetic group in cases and controls < 100.

Replication of association

A: Little between-study heterogeneity — $I^2 < 25\%$.

B: Moderate between-study heterogeneity — I^2 between 25% and 50%.

C: Large between-study heterogeneity — $I^2 > 50\%$.

Qualitative epidemiologic considerations about the presence of heterogeneity and potential explanation for heterogeneity would need to be taken into account in judging replication. It may be reasonable to grade as A on this criterion for associations with moderate or high heterogeneity with an extensive replication record such as associations identified by GWAS or large GWAS meta-analysis from collaborative studies.

Protection from bias

A: No observable bias and bias was unlikely to explain the presence of the association. B: No obvious bias may affect the presence of the association, but there is considerable missing information on the identification of evidence. C: Bias is demonstrable or is likely to explain the presence of the association. The Venice criteria include an extensive checklist for sources of bias in different settings. The checklist has different considerations depending on whether the evidence comes from retrospective meta-analyses of published data or prospective GWAS and replication studies from collaborative consortia with harmonization of data collection and analysis.

General checks for bias that have been adopted for meta-analysis are: (1) Association lost with exclusion of first study; (2) Association lost with exclusion of studies deviated from HWE; (3) Small effect size of association (i.e., $0.87 < OR < 1.15$); (4) Evidence of publication bias ($p < 0.10$ in Begg's test); (5) Evidence of small-study effect ($p < 0.10$ in Egger's test); (6) Evidence is presented for an excess of individual studies with significant findings ($p < 0.10$ in significant bias test).

Method for false positive report probability (FPRP) test

A prior probability of 0.05 and a false positive report probability (FPRP) cut-off value of 0.2 in FPRP assay should be performed to detect the potential false positive results among statistical associations and assess whether these associations should be excluded, as Wacholder et al. recommended [(4)]. If the calculated FPRP value was below the prespecified noteworthiness value of 0.2, we would consider the association noteworthy, indicating the association might be true [(4)]. The true evidence was graded by FPRP value: <0.05, 0.05 - 0.2, >0.2, indicating strong, moderate, or weak, respectively. Cumulative evidence could be upgraded from moderate to strong or from weak to moderate based on a strong FPRP (<0.05). Otherwise, cumulative evidence could be downgraded from strong to moderate or from moderate to weak based on weak FPRP. The cumulative epidemiological evidence remained the same with the result of the Venice criteria for associations with moderate evidence of FPRP. We used the Excel calculator provided by Wacholder *et al*. to calculate the FPRP value and statistical power (>0.2) [(4)].

Supplementary Results:

Null association between variant in TERT-CLPTM1L genes and risk of cancer or noncancerous disease

In our study, 13 SNPs (*TERT* MNS16A, rs13167280, rs2075786, rs2735940, rs2736100, rs2736109, rs2853669, rs2853677, rs2853690, rs7712562, rs2735940, rs2736098, rs4246742 and *TERT* - *CLPTM1L* rs4635969) had no association with risk of five cancer risk in additive model (breast, gastric, hepatocellular, lung and pancreatic cancer). Of these, 10 SNPs had no association with breast cancer risk, as follow: *TERT* MNS16A (Caucasians: OR = 1.065, 95% CI: 0.845-1.341, p = 0.595), *TERT* rs13167280 (Caucasians: OR = 0.963, 95% CI: 0.888-1.043, p = 0.349), *TERT* rs2075786 (Caucasians: OR = 0.996, 95% CI: 0.942-1.054, p = 0.902), *TERT* rs2735940 (Overall: OR = 0.978, 95% CI: 0.913-1.048, p = 0.534; Caucasians: OR = 1.006, 95% CI: 0.957-1.059, p = 0.803), *TERT* rs2736100 (Caucasians: OR =1.062, 95% CI: 0.946-1.192, p = 0.309), *TERT* rs2736109 (Overall: OR = 0.987, 95% CI: 0.922-1.056, p = 0.704; Caucasians: OR = 0.995, 95% CI: 0.957-1.035, p = 0.809; Asians: OR = 1.023, 95% CI: 0.841-1.246, p = 0.817), *TERT* rs2853669 (Caucasians: OR = 1.006, 95% CI: 0.942-1.074, p = 0.870), *TERT* rs2853677 (Caucasians: OR = 1.036, 95% CI: 0.980-1.095, p = 0.221), *TERT* rs2853690 (Caucasians: OR = 0.541, 95% CI: 0.905-1.054, p = 0.541), *TERT* rs7712562 (Caucasians: OR = 1.035, 95% CI: 0.927-1.156, p = 0.540). Moreover, *TERT* rs2735940 had null associations with gastric cancer (Overall: OR = 1.302, 95% CI: 0.689-2.460, p = 0.416; Asians: OR = 1.748, 95% CI: 0.894-3.419, p = 0.103). No association was found for *TERT* rs2736098 with risk of hepatocellular carcinoma in Asians (OR = 1.211, 95% CI: 0.948-1.548, p = 0.125), rs4246742 with risk of lung cancer in Asians (OR = 1.133, 95% CI: 0.875-1.467, p = 0.343), and *TERT/CLPTM1L* rs4635969 with pancreatic cancer (OR = 1.026, 95% CI: 0.943-1.117, p = 0.547). Interestingly, we found that eight SNPs had no association with breast cancer with at least 5,000 case and 5,000 controls in additive model, which offers over 98% statistical power to detect an OR of 1.15 under the additive model for a variant with MAF 0.20 and 86% power to detect an OR of 1.15 under the additive model for a variant with MAF 0.10, Type 1 error 0.05. Therefore, further research on these eight SNPs for breast cancer with a similar sample size may not yield fruitful results.

References:

1. Ioannidis-John PA, Boffetta P, Little J, O'Brien TR, Uitterlinden AG, Vineis P, Balding DJ, Chokkalingam A, Dolan SM, Flanders WD, Higgins-Julian PT, McCarthy MI, McDermott DH, Page GP, Rebbeck TR, Seminara D, Khoury MJ. Assessment of cumulative evidence on genetic associations: interim guidelines. Int J Epidemiol **2008**;37:120-32.
2. Khoury MJ, Bertram L, Boffetta P, Butterworth AS, Chanock SJ, Dolan SM, et al. Genome-wide association studies, field synopses, and the development of the knowledge base on genetic variation and human diseases. Am J Epidemiol 2009;170:269-79.
3. Cui HJ, Tang MS, Zhang M, Liu SS, Chen SY, Zeng ZQ, et al. Variants in the PSCA gene associated with risk of cancer and nonneoplastic diseases: systematic research synopsis, meta-analysis and epidemiological evidence. Carcinogenesis 2019;40:70-83.
4. Wacholder S, Chanock S, Garcia-Closas M, Ghormli LE, Rothman N. Assessing the probability that a positive report is false: an approach for molecular epidemiology studies. J Natl Cancer Inst 2004;96:434-42.
