# Supplementary material for: Cumulative Evidence for Relationships Between Multiple Variants in the TERT and CLPTM1L Region and Risk of Cancer and Non-Cancer Disease
Source: Front Oncol. 2022 Jun 30;12:946039. doi: 10.3389/fonc.2022.946039 (PMC9279858; doi:10.3389/fonc.2022.946039)
Supplement: Supplementary file 3 [file DataSheet_3.pdf]

Supplementary Table 2. Characteristics of the included articles.

Ref Number	First author, year	Disease	Disease Type	Country/ region	Ethnicity	Study design	Gene name	variant	Allelic	Case	control	Sample size
1	Savage SA, 2007	Breast cancer		Poland	Caucasian	Case-control study	TERT	rs2736109 G/A	G/A	1995	2296	4291
1	Savage SA, 2007	Breast cancer		Poland	Caucasian	Case-control study	TERT	rs2735940 A/G	A/G	1995	2296	4291
1	Savage SA, 2007	Breast cancer		Poland	Caucasian	Case-control study	TERT	rs7712562 A/C	A/C	1995	2296	4291
1	Savage SA, 2007	Breast cancer		Poland	Caucasian	Case-control study	TERT	rs2853669 T/C	T/C	1995	2296	4291
1	Savage SA, 2007	Breast cancer		Poland	Caucasian	Case-control study	TERT	rs2736098 C/T	C/T	1995	2296	4291
1	Savage SA, 2007	Breast cancer		Poland	Caucasian	Case-control study	TERT	rs2853677 G/A	G/A	1995	2296	4291
1	Savage SA, 2007	Breast cancer		Poland	Caucasian	Case-control study	TERT	rs13167280 G/A	G/A	1995	2296	4291
1	Savage SA, 2007	Breast cancer		Poland	Caucasian	Case-control study	TERT	rs2075786 A/G	A/G	1995	2296	4291
1	Savage SA, 2007	Breast cancer		Poland	Caucasian	Case-control study	TERT	rs2853690 G/T	G/T	1995	2296	4291
2	Wang Y, 2008	Breast cancer		China	Caucasian	Case-control study	TERT	MNS16A L/S	L/S	1006	1095	2101
3	Mushiroda T,2008	Idiopathic pulmonary fibrosis (IPF)		Japan	Asian	GWAS	TERT	rs2736100 C/A	C/A	159	934	1093
3	Mushiroda T,2008	Idiopathic pulmonary fibrosis (IPF)		Japan	Asian	Case-control study	TERT	rs2736100 C/A	C/A	83	535	618

4	Wang YF, 2008	Lung cancer	Miscellany	United Kingdom (UK)	Caucasian	GWAS	CLPTM1L	rs401681 C/T	C/T	1952	1438	3390
4	Wang YF, 2008	Lung cancer	NSCLC	USA	Caucasian	GWAS	CLPTM1L	rs401681 C/T	C/T	1154	1137	2291
4	Wang YF, 2008	Lung cancer	Miscellany	Europe countries (Czech Republic, Hungary, Poland, Romania, Russia and Slovakia)	Caucasian	GWAS	CLPTM1L	rs401681 C/T	C/T	1989	2625	4614
4	Wang YF, 2008	Lung cancer	Miscellany	United Kingdom (UK)	Caucasian	GWAS	TERT/CLPTM1L	rs4975616 G/A	G/A	1952	1438	3390
4	Wang YF, 2008	Lung cancer	NSCLC	USA	Caucasian	GWAS	TERT/CLPTM1L	rs4975616 G/A	G/A	1154	1137	2291
4	Wang YF, 2008	Lung cancer	Miscellany	Europe countries (Czech Republic, Hungary, Poland, Romania, Russia and Slovakia)	Caucasian	GWAS	TERT/CLPTM1L	rs4975616 G/A	G/A	1989	2625	4614
4	Wang YF, 2008	Lung cancer	Miscellany	United Kingdom (UK)	Caucasian	GWAS	CLPTM1L	rs31489 C/A	C/A	1952	1438	3390

4	Wang YF, 2008	Lung cancer	NSCLC	USA	Caucasian	GWAS	CLPTM1L	rs31489 C/A	C/A	1154	1137	2291
4	Wang YF, 2008	Lung cancer	Miscellany	Europe countries (Czech Republic, Hungary, Poland, Romania, Russia and Slovakia)	Caucasian	GWAS	CLPTM1L	rs31489 C/A	C/A	1989	2625	4614
4	Wang YF, 2008	Lung cancer	Small cell carcinoma	United Kingdom (UK)	Caucasian	GWAS	CLPTM1L	rs401681 C/T	C/T	1028	1438	2466
4	Wang YF, 2008	Lung cancer	NSCLC	United Kingdom (UK)	Caucasian	GWAS	CLPTM1L	rs401681 C/T	C/T	3316	1438	4754
4	Wang YF, 2008	Lung cancer	NSCLC (Squamous cell carcinoma)	United Kingdom (UK)	Caucasian	GWAS	CLPTM1L	rs401681 C/T	C/T	1513	1438	2951
4	Wang YF, 2008	Lung cancer	NSCLC (Adenocarcinoma)	United Kingdom (UK)	Caucasian	GWAS	CLPTM1L	rs401681 C/T	C/T	939	1438	2377
4	Wang YF, 2008	Lung cancer	NSCLC (Squamous cell carcinoma)	USA	Caucasian	GWAS	CLPTM1L	rs401681 C/T	C/T	306	1137	1443
4	Wang YF, 2008	Lung cancer	NSCLC (Adenocarcinoma)	USA	Caucasian	GWAS	CLPTM1L	rs401681 C/T	C/T	620	1137	1757
5	Jin GF, 2009	Lung cancer	NSCLC	China	Asian	Case-control study	TERT	rs2736100 C/A	C/A	1221	1344	2565
5	Jin GF, 2009	Lung cancer	NSCLC	China	Asian	Case-control study	CLPTM1L	rs402710 C/T	C/T	1221	1344	2565

6	Varadi V, 2009	Breast cancer		Poland	Caucasian	Case-control study	TERT	rs2853669 T/C	T/C	768	460	1228
6	Varadi V, 2009	Breast cancer		Sweden	Caucasian	Case-control study	TERT	rs2853669 T/C	T/C	766	1519	2285
7	Zienolddiny S, 2009	Lung cancer	Miscellany	Norway	Caucasian	Case-control study	CLPTM1L	rs402710 C/T	C/T	365	440	805
7	Zienolddiny S, 2009	Lung cancer	Miscellany	Norway	Caucasian	Case-control study	CLPTM1L	rs401681 C/T	C/T	365	440	805
8	Shete S, 2009	Glioma		France	Caucasian	Case-control study	TERT	rs2736100 C/A	C/A	1392	1602	2994
8	Shete S, 2009	Glioma		France	Caucasian	Case-control study	TERT	rs2853676 T/C	T/C	1392	1602	2994
8	Shete S, 2009	Glioma		Germany	Caucasian	Case-control study	TERT	rs2736100 C/A	C/A	504	573	1077
8	Shete S, 2009	Glioma		Germany	Caucasian	Case-control study	TERT	rs2853676 T/C	T/C	504	573	1077
8	Shete S, 2009	Glioma		Sweden	Caucasian	Case-control study	TERT	rs2736100 C/A	C/A	649	778	1427
8	Shete S, 2009	Glioma		Sweden	Caucasian	Case-control study	TERT	rs2853676 T/C	T/C	649	778	1427
8	Shete S, 2009	Glioma		United Kingdom (UK)	Caucasian	GWAS	TERT	rs2736100 C/A	C/A	631	1434	2065
8	Shete S, 2009	Glioma		United Kingdom (UK)	Caucasian	GWAS	TERT	rs2853676 T/C	T/C	631	1434	2065
8	Shete S, 2009	Glioma		USA	Caucasian	GWAS	TERT	rs2736100 C/A	C/A	1247	2236	3483
8	Shete S, 2009	Glioma		USA	Caucasian	GWAS	TERT	rs2853676 T/C	T/C	1247	2236	3483
9	Broderick P, 2009	Lung cancer	Miscellany	United Kingdom (UK)	Caucasian	GWAS	TERT/CLPTM1L	rs4975616 G/A	G/A	4019	907	4926
9	Broderick P, 2009	Lung cancer	Miscellany	United Kingdom (UK)	Caucasian	GWAS	TERT	rs2736100 C/A	C/A	4019	907	4926
10	Varadi V, 2009	Breast cancer		Sweden	Caucasian	Case-control study	TERT	rs2853669 T/C	T/C	782	1559	2341
11	Choi JE, 2009	Lung cancer	Miscellany	Korea	Asian	Case-control study	TERT	rs2736098 C/T	C/T	239	553	792
12	Landi MT, 2009	Lung cancer	Miscellany	multiple	Caucasian	GWAS	CLPTM1L	rs31489 C/A	C/A	5739	5848	11587

				countries								
13	Wang YF, 2010	Lung cancer	Miscellany	United Kingdom (UK)	Caucasian	Case-control study	TERT	rs2736100 C/A	C/A	239	553	792
13	Wang YF, 2010	Lung cancer	Miscellany	United Kingdom (UK)	Caucasian	Case-control study	TERT/CLPTM1L	rs4975616 G/A	G/A	239	553	792
13	Wang YF, 2010	Lung cancer	NSCLC	United Kingdom (UK)	Caucasian	Case-control study	TERT	rs2736100 C/A	C/A	200	553	753
13	Wang YF, 2010	Lung cancer	NSCLC	United Kingdom (UK)	Caucasian	Case-control study	TERT/CLPTM1L	rs4975616 G/A	G/A	200	553	753
13	Wang YF, 2010	Lung cancer	NSCLC (Adenocarcinoma)	United Kingdom (UK)	Caucasian	Case-control study	TERT	rs2736100 C/A	C/A	112	553	665
13	Wang YF, 2010	Lung cancer	NSCLC (Squamous cell carcinoma)	United Kingdom (UK)	Caucasian	Case-control study	TERT	rs2736100 C/A	C/A	48	553	601
14	Shen J, 2010	Breast cancer		multiple countries	Caucasian	Case-control study	TERT	MNS16A L/S	L/S	1067	1110	2177
14	Shen J, 2010	Breast cancer		multiple countries	Caucasian	Case-control study	TERT	rs2075786 A/G	A/G	1067	1110	2177
14	Shen J, 2010	Breast cancer		multiple countries	Caucasian	Case-control study	TERT	rs2853690 G/T	G/T	1067	1110	2177
14	Shen J, 2010	Breast cancer		multiple countries	Caucasian	Case-control study	TERT	rs2736109 G/A	G/A	1067	1110	2177
14	Shen J, 2010	Breast cancer		multiple countries	Caucasian	Case-control study	TERT	rs2735940 A/G	A/G	1067	1110	2177
14	Shen J, 2010	Breast cancer		multiple countries	Caucasian	Case-control study	TERT	rs7712562 A/C	A/C	1067	1110	2177

14	Shen J, 2010	Breast cancer		multiple countries	Caucasian	Case-control study	TERT	rs2853677 G/A	G/A	1067	1110	2177
14	Shen J, 2010	Breast cancer		multiple countries	Caucasian	Case-control study	TERT	rs13167280 G/A	G/A	1067	1110	2177
14	Shen J, 2010	Breast cancer		multiple countries	Caucasian	Case-control study	TERT	rs2853669 T/C	T/C	1067	1110	2177
15	Truong T, 2010	Lung cancer	Miscellany	multiple countries	Caucasian	Case-control study	TERT	rs2736100 C/A	C/A	9951	12828	22779
15	Truong T, 2010	Lung cancer	Miscellany	multiple countries	Caucasian	Case-control study	CLPTM1L	rs402710 C/T	C/T	9951	12828	22779
15	Truong T, 2010	Lung cancer	Miscellany	multiple countries	Asian	Case-control study	TERT	rs2736100 C/A	C/A	1694	2126	3820
15	Truong T, 2010	Lung cancer	Miscellany	multiple countries	Asian	Case-control study	CLPTM1L	rs402710 C/T	C/T	1694	2126	3820
16	Hsiung CA, 2010	Lung cancer	NSCLC (Adenocarcinoma)	China	Asian	GWAS	TERT	rs2736100 C/A	C/A	584	585	1169
16	Hsiung CA, 2010	Lung cancer	Miscellany	China	Asian	Case-control study	TERT	rs2736100 C/A	C/A	610	560	1170
16	Hsiung CA, 2010	Lung cancer	NSCLC (Adenocarcinoma)	China	Asian	Case-control study	TERT	rs2736100 C/A	C/A	407	560	967
16	Hsiung CA, 2010	Lung cancer	Miscellany	China	Asian	Case-control study	TERT	rs2736100 C/A	C/A	287	287	574
16	Hsiung CA, 2010	Lung cancer	NSCLC (Adenocarcinoma)	China	Asian	Case-control study	TERT	rs2736100 C/A	C/A	188	287	475
16	Hsiung CA, 2010	Lung cancer	Miscellany	Korea	Asian	Case-control study	TERT	rs2736100 C/A	C/A	259	293	552
16	Hsiung CA, 2010	Lung cancer	NSCLC (Adenocarcinoma)	Korea	Asian	Case-control study	TERT	rs2736100 C/A	C/A	197	293	490
16	Hsiung CA, 2010	Lung cancer	Miscellany	China	Asian	Cohort sytudy	TERT	rs2736100 C/A	C/A	209	213	422
16	Hsiung CA, 2010	Lung cancer	NSCLC (Adenocarcinoma)	China	Asian	Cohort sytudy	TERT	rs2736100 C/A	C/A	94	213	307

16	Hsiung CA, 2010	Lung cancer	Miscellany	China	Asian	Case-control study	TERT	rs2736100 C/A	C/A	207	207	414
16	Hsiung CA, 2010	Lung cancer	NSCLC (Adenocarcinoma)	China	Asian	Case-control study	TERT	rs2736100 C/A	C/A	89	207	296
16	Hsiung CA, 2010	Lung cancer	Miscellany	Korea	Asian	Case-control study	TERT	rs2736100 C/A	C/A	121	119	240
16	Hsiung CA, 2010	Lung cancer	NSCLC (Adenocarcinoma)	Korea	Asian	Case-control study	TERT	rs2736100 C/A	C/A	103	119	222
16	Hsiung CA, 2010	Lung cancer	Miscellany	Korea	Asian	Case-control study	TERT	rs2736100 C/A	C/A	95	87	182
16	Hsiung CA, 2010	Lung cancer	NSCLC (Adenocarcinoma)	Korea	Asian	Case-control study	TERT	rs2736100 C/A	C/A	66	87	153
16	Hsiung CA, 2010	Lung cancer	Miscellany	multiple countries	Asian	Case-control study	TERT	rs4246742 T/A	T/A	2768	3100	5868
16	Hsiung CA, 2010	Lung cancer	Miscellany	multiple countries	Asian	Case-control study	TERT	rs2242652 G/A	G/A	2768	3100	5868
16	Hsiung CA, 2010	Lung cancer	Miscellany	multiple countries	Asian	Case-control study	CLPTM1L	rs402710 C/T	C/T	2768	3100	5868
16	Hsiung CA, 2010	Lung cancer	NSCLC (Adenocarcinoma)	multiple countries	Asian	Case-control study	CLPTM1L	rs402710 C/T	C/T	2003	3100	3103
17	Miki D, 2010	Lung cancer	NSCLC (Adenocarcinoma)	Japan	Asian	GWAS	TERT	rs2736100 C/A	C/A	1004	1900	2904
17	Miki D, 2010	Lung cancer	NSCLC (Adenocarcinoma)	Japan	Asian	Replication stage	TERT	rs2736100 C/A	C/A	525	7678	8203
17	Miki D, 2010	Lung cancer	NSCLC (Adenocarcinoma)	Japan	Asian	Replication stage	TERT	rs2736100 C/A	C/A	569	1470	2039
18	Yoon KA, 2010	Lung cancer	NSCLC	Korea	Asian	GWAS	TERT	rs2736100 C/A	C/A	1425	3011	4436
18	Yoon KA, 2010	Lung cancer	NSCLC	Korea	Asian	GWAS	TERT	rs2736100 C/A	C/A	1425	3011	4436
18	Yoon KA, 2010	Lung cancer	NSCLC	Korea	Asian	GWAS	TERT	rs2736100 C/A	C/A	1425	3011	4436

18	Yoon KA, 2010	Lung cancer	NSCLC	Korea	Asian	GWAS	CLPTM1L	rs402710 C/T	C/T	1425	3011	4436
18	Yoon KA, 2010	Lung cancer	NSCLC	Korea	Asian	GWAS	CLPTM1L	rs402710 C/T	C/T	1425	3011	4436
18	Yoon KA, 2010	Lung cancer	NSCLC	Korea	Asian	GWAS	CLPTM1L	rs402710 C/T	C/T	1425	3011	4436
18	Yoon KA, 2010	Lung cancer	NSCLC	Korea	Asian	GWAS	CLPTM1L	rs401681 C/T	C/T	1425	3011	4436
18	Yoon KA, 2010	Lung cancer	NSCLC	Korea	Asian	GWAS	CLPTM1L	rs401681 C/T	C/T	1425	3011	4436
18	Yoon KA, 2010	Lung cancer	NSCLC	Korea	Asian	GWAS	CLPTM1L	rs401681 C/T	C/T	1425	3011	4436
19	Gago Dominguez M, 2011	Bladder cancer		USA	Caucasian	Case-control study	CLPTM1L	rs401681 C/T	C/T	498	588	1086
19	Gago Dominguez M, 2011	Bladder cancer		China	Asian	Case-control study	CLPTM1L	rs401681 C/T	C/T	506	530	1036
19	Gago Dominguez M, 2011	Bladder cancer		USA	Caucasian	Case-control study	CLPTM1L	rs402710 C/T	C/T	498	588	1086
19	Gago Dominguez M, 2011	Bladder cancer		China	Asian	Case-control study	CLPTM1L	rs402710 C/T	C/T	506	530	1036
19	Gago Dominguez M, 2011	Bladder cancer		USA	Caucasian	Case-control study	TERT	rs2736098 C/T	C/T	498	588	1086
19	Gago Dominguez M, 2011	Bladder cancer		China	Asian	Case-control study	TERT	rs2736098 C/T	C/T	506	530	1036
19	Gago Dominguez M, 2011	Bladder cancer		USA	Caucasian	Case-control study	TERT	rs2736100 C/A	C/A	498	588	1086
19	Gago Dominguez M, 2011	Bladder cancer		China	Asian	Case-control study	TERT	rs2736100 C/A	C/A	506	530	1036
20	Nan HM, 2011	Skin cancer	melanoma	United Kingdom (UK)	Caucasian	Nested case-control study	CLPTM1L	rs401681 C/T	C/T	218	870	1088
21	Chen HY, 2011	Glioma		China	Asian	Case-control study	TERT	rs2736100 C/A	C/A	976	1057	2033
21	Chen HY, 2011	Glioma		China	Asian	Case-control study	TERT	rs2853676 T/C	T/C	976	1057	2033
22	Kohno T, 2011	Lung cancer	NSCLC (Squamous cell carcinoma)	Japan	Asian	Case-control study	TERT	rs2736100 C/A	C/A	377	325	702
23	Hu ZB, 2011	Lung cancer	Miscellany	China	Asian	GWAS	CLPTM1L	rs465498 A/G	A/G	2331	3077	5408

23	Hu ZB, 2011	Lung cancer	Miscellany	China	Asian	Case-control study	CLPTM1L	rs465498 A/G	A/G	2283	2243	4526
23	Hu ZB, 2011	Lung cancer	Miscellany	China	Asian	Case-control study	CLPTM1L	rs465498 A/G	A/G	4030	4166	8196
23	Hu ZB, 2011	Lung cancer	Miscellany	China	Asian	GWAS	TERT	rs2736100 C/A	C/A	2331	3077	5408
23	Hu ZB, 2011	Lung cancer	Miscellany	China	Asian	Case-control study	TERT	rs2736100 C/A	C/A	2283	2243	4526
23	Hu ZB, 2011	Lung cancer	Miscellany	China	Asian	Case-control study	TERT	rs2736100 C/A	C/A	4030	4166	8196
24	Pande M, 2011	Lung cancer	Miscellany	multiple countries	Caucasian	Case-control study	CLPTM1L	rs31489 C/A	C/A	1681	1635	3316
25	Beesley J, 2011	Breast cancer		Austria	Caucasian	Case-control study	TERT	rs2736109 G/A	G/A	1118	934	2052
25	Beesley J, 2011	Breast cancer		Germany	Caucasian	Case-control study	TERT	rs2736109 G/A	G/A	445	554	999
25	Beesley J, 2011	Breast cancer		Germany	Caucasian	Case-control study	TERT	rs2736109 G/A	G/A	2387	4945	7332
25	Beesley J, 2011	Breast cancer		Thailand	Others	Case-control study	TERT	rs2736109 G/A	G/A	327	567	894
26	Jaworowska E, 2011	Lung cancer	Miscellany	Poland	Caucasian	Case-control study	TERT	rs2736100 C/A	C/A	874	1061	1935
26	Jaworowska E, 2011	Bladder cancer		Poland	Caucasian	Case-control study	TERT	rs2736100 C/A	C/A	450	1061	1511
26	Jaworowska E, 2011	Lung cancer	Miscellany	Poland	Caucasian	Case-control study	TERT	rs2736098 C/T	C/T	874	1061	1935
26	Jaworowska E, 2011	Bladder cancer		Poland	Caucasian	Case-control study	TERT	rs2736098 C/T	C/T	450	1061	1511
26	Jaworowska E, 2011	Lung cancer	Miscellany	Poland	Caucasian	Case-control study	CLPTM1L	rs402710 C/T	C/T	874	1061	1935
26	Jaworowska E, 2011	Bladder cancer		Poland	Caucasian	Case-control study	CLPTM1L	rs402710 C/T	C/T	450	1061	1511
27	Rizzato C, 2011	Pancreatic cancer		Germany, United Kingdom	Caucasian	Case-control study	TERT/CLPTM1L	rs4635969 G/A	G/A	114	136	250
27	Rizzato C, 2011	Pancreatic cancer		Germany, United Kingdom	Caucasian	Case-control study	TERT/CLPTM1L	rs4635969 G/A	G/A	576	1141	1717

27	Rizzato C, 2011	Pancreatic cancer		Germany, United Kingdom	Caucasian	Case-control study	CLPTM1L	rs401681 C/T	C/T	114	136	250
27	Rizzato C, 2011	Pancreatic cancer		Germany, United Kingdom	Caucasian	Case-control study	CLPTM1L	rs401681 C/T	C/T	576	1141	1717
28	Pellatt AJ, 2013	Colorectal cancer	Miscellany	USA	Caucasian	Case-control study	TERT	rs2736100 C/A	C/A	2309	2915	5224
29	Chen XF, 2012	Lung cancer	Miscellany	China	Asian	Case-control study	CLPTM1L	rs402710 C/T	C/T	196	229	425
29	Chen XF, 2012	Lung cancer	NSCLC (Adenocar cinoma)	China	Asian	Case-control study	CLPTM1L	rs402710 C/T	C/T	96	229	325
29	Chen XF, 2012	Lung cancer	Miscellany	China	Asian	Case-control study	CLPTM1L	rs401681 C/T	C/T	196	229	425
29	Chen XF, 2012	Lung cancer	NSCLC (Adenocar cinoma)	China	Asian	Case-control study	CLPTM1L	rs401681 C/T	C/T	96	229	325
29	Chen XF, 2012	Lung cancer	NSCLC (Squamous cell carcinoma)	China	Asian	Case-control study	CLPTM1L	rs401681 C/T	C/T	44	229	273
29	Chen XF, 2012	Lung cancer	Small cell carcinoma	China	Asian	Case-control study	CLPTM1L	rs401681 C/T	C/T	16	229	245
29	Chen XF, 2012	Lung cancer	Miscellany	China	Asian	Case-control study	TERT	rs2736100 C/A	C/A	196	229	425
29	Chen XF, 2012	Lung cancer	NSCLC (Adenocar cinoma)	China	Asian	Case-control study	TERT	rs2736100 C/A	C/A	96	229	325
29	Chen XF, 2012	Lung cancer	NSCLC (Squamous cell carcinoma)	China	Asian	Case-control study	TERT	rs2736100 C/A	C/A	44	229	273
30	Bae EY, 2012	Lung cancer	Miscellany	Korea	Asian	Case-control study	TERT	rs2736100 C/A	C/A	1094	1000	2094
30	Bae EY, 2012	Lung cancer	Miscellany	Korea	Asian	Case-control study	CLPTM1L	rs402710 C/T	C/T	1094	1000	2094
30	Bae EY, 2012	Lung cancer	Miscellany	Korea	Asian	Case-control study	CLPTM1L	rs401681 C/T	C/T	1094	1000	2094

30	Bae EY, 2012	Lung cancer	Miscellany	Korea	Asian	Case-control study	CLPTM1L	rs31489 C/A	C/A	1094	1000	2094
31	Ito H, 2012	Lung cancer	Miscellany	Japan	Asian	Case-control study	TERT	rs2736100 C/A	C/A	716	716	1432
31	Ito H, 2012	Lung cancer	Miscellany	Japan	Asian	Case-control study	CLPTM1L	rs402710 C/T	C/T	716	716	1432
32	Hofer P, 2012	Colorectal cancer	Miscellany	Austria	Caucasian	Case-control study	TERT	rs2736100 C/A	C/A	142	1793	1935
33	Ma ZC, 2013	Bladder cancer		China	Asian	Case-control study	TERT	rs2736100 C/A	C/A	184	962	1146
33	Ma ZC, 2013	Bladder cancer		China	Asian	Case-control study	TERT	rs2736098 C/T	C/T	184	962	1146
33	Ma ZC, 2013	Bladder cancer		China	Asian	Case-control study	CLPTM1L	rs401681 C/T	C/T	184	962	1146
34	Kinnersley B, 2012	Colorectal cancer	Miscellany	Austria	Caucasian	GWAS	TERT	rs2736100 C/A	C/A	379	1703	2082
34	Kinnersley B, 2012	Colorectal cancer	Miscellany	USA	Caucasian	GWAS	TERT	rs2736100 C/A	C/A	1175	999	2174
34	Kinnersley B, 2012	Colorectal cancer	Miscellany	USA	Caucasian	GWAS	TERT	rs2736100 C/A	C/A	796	2234	3030
34	Kinnersley B, 2012	Colorectal cancer	Miscellany	Europe	Caucasian	GWAS	TERT	rs2736100 C/A	C/A	890	900	1790
34	Kinnersley B, 2012	Colorectal cancer	Miscellany	Scotland	Caucasian	GWAS	TERT	rs2736100 C/A	C/A	973	998	1971
34	Kinnersley B, 2012	Colorectal cancer	Miscellany	United Kingdom (UK)	Caucasian	GWAS	TERT	rs2736100 C/A	C/A	1794	2686	4480
34	Kinnersley B, 2012	Colorectal cancer	Miscellany	Europe	Caucasian	Case-control study	TERT	rs2736100 C/A	C/A	10488	7137	17625
35	Zagouri F, 2012	Breast cancer		Greece	Caucasian	Case-control study	TERT	MNS16A L/S	L/S	113	124	237
36	Wang H, 2013	Lung cancer	Miscellany	China	Asian	Case-control study	CLPTM1L	rs401681 C/T	C/T	492	486	978
36	Wang H, 2013	Lung cancer	NSCLC	China	Asian	Case-control study	CLPTM1L	rs401681 C/T	C/T	393	486	879
36	Wang H, 2013	Lung cancer	Small cell carcinoma	China	Asian	Case-control study	CLPTM1L	rs401681 C/T	C/T	99	486	585
37	Zhao ZH, 2013	Lung cancer	Miscellany	China	Asian	Case-control study	CLPTM1L	rs31489 C/A	C/A	784	782	1566

38	Zhang C, 2013	Hepatocellular carcinoma		China	Asian	Case-control study	TERT	rs2736098 C/T	C/T	400	400	800
39	Myneni AA, 2013	Lung cancer	Miscellany	China	Asian	Case-control study	TERT	rs2736100 C/A	C/A	399	466	865
39	Myneni AA, 2013	Lung cancer	Miscellany	China	Asian	Case-control study	CLPTM1L	rs401681 C/T	C/T	399	466	865
40	Lan Q, 2013	Lung cancer	Miscellany	China	Asian	Nested case-control study	TERT	rs2736100 C/A	C/A	215	215	430
41	Pellatt AJ, 2013	Breast cancer		Poland	Caucasian	Case-control study	TERT	rs2736109 G/A	G/A	1995	2296	4291
41	Pellatt AJ, 2013	Breast cancer		Poland	Caucasian	Case-control study	TERT	rs2735940 A/G	A/G	1995	2296	4291
41	Pellatt AJ, 2013	Breast cancer		Poland	Caucasian	Case-control study	TERT	rs7712562 A/C	A/C	1995	2296	4291
41	Pellatt AJ, 2013	Breast cancer		Poland	Caucasian	Case-control study	TERT	rs2853669 T/C	T/C	1995	2296	4291
41	Pellatt AJ, 2013	Breast cancer		Poland	Caucasian	Case-control study	TERT	rs2736098 C/T	C/T	1995	2296	4291
41	Pellatt AJ, 2013	Breast cancer		Poland	Caucasian	Case-control study	TERT	rs2853677 G/A	G/A	1995	2296	4291
41	Pellatt AJ, 2013	Breast cancer		Poland	Caucasian	Case-control study	TERT	rs13167280 G/A	G/A	1995	2296	4291
41	Pellatt AJ, 2013	Breast cancer		Poland	Caucasian	Case-control study	TERT	rs2075786 A/G	A/G	1995	2296	4291
41	Pellatt AJ, 2013	Breast cancer		Poland	Caucasian	Case-control study	TERT	rs2853690 G/T	G/T	1995	2296	4291
42	Ke JT, 2013	Lung cancer	Miscellany	China	Asian	Case-control study	CLPTM1L	rs401681 C/T	C/T	611	1062	1673
42	Ke JT, 2013	Lung cancer	NSCLC	China	Asian	Case-control study	CLPTM1L	rs401681 C/T	C/T	427	1062	1489
43	Li C, 2013	Lung cancer	Miscellany	China	Asian	Case-control study	TERT	rs2736098 C/T	C/T	501	576	1077
43	Li C, 2013	Lung cancer	NSCLC (Adenocarcinoma)	China	Asian	Case-control study	TERT	rs2736098 C/T	C/T	334	576	910
43	Li C, 2013	Lung cancer	NSCLC (Squamous cell	China	Asian	Case-control study	TERT	rs2736098 C/T	C/T	90	576	666

			carcinoma)									
43	Li C, 2013	Lung cancer	Miscellany	China	Asian	Case-control study	CLPTM1L	rs401681 C/T	C/T	501	576	1077
43	Li C, 2013	Lung cancer	NSCLC (Adenocarcinoma)	China	Asian	Case-control study	CLPTM1L	rs401681 C/T	C/T	334	576	910
43	Li C, 2013	Lung cancer	NSCLC (Squamous cell carcinoma)	China	Asian	Case-control study	CLPTM1L	rs401681 C/T	C/T	90	576	666
44	Lizuka T, 2013	Gastric cancer		Japan	Asian	Case-control study	TERT	rs2735940 A/G	A/G	177	591	768
44	Lizuka T, 2013	Breast cancer		Japan	Asian	Case-control study	TERT	rs2735940 A/G	A/G	68	591	659
45	Jin TB, 2013	Glioma		China	Asian	Case-control study	TERT	rs2853676 T/C	T/C	433	463	896
46	de Mello, 2013	Lung cancer	NSCLC	China	Asian	Case-control study	CLPTM1L	rs31489 C/A	C/A	144	144	288
46	de Mello, 2013	Lung cancer	NSCLC	China	Asian	Case-control study	CLPTM1L	rs401681 C/T	C/T	144	144	288
46	de Mello, 2013	Lung cancer	NSCLC	China	Asian	Case-control study	CLPTM1L	rs402710 C/T	C/T	144	144	288
47	Sun Y, 2013	Lung cancer	NSCLC	China	Asian	Case-control study	CLPTM1L	rs401681 C/T	C/T	200	200	400
47	Sun Y, 2013	Lung cancer	NSCLC (Adenocarcinoma)	China	Asian	Case-control study	CLPTM1L	rs401681 C/T	C/T	145	200	345
47	Sun Y, 2013	Lung cancer	NSCLC (Squamous cell carcinoma)	China	Asian	Case-control study	CLPTM1L	rs401681 C/T	C/T	55	200	255
47	Sun Y, 2013	Lung cancer	NSCLC	China	Asian	Case-control study	TERT/CLPTM1L	rs4975616 G/A	G/A	200	200	400
47	Sun Y, 2013	Lung cancer	NSCLC (Adenocarcinoma)	China	Asian	Case-control study	CLPTM1L	rs401681 C/T	C/T	145	200	345
47	Sun Y, 2013	Lung cancer	NSCLC (Squamous cell carcinoma)	China	Asian	Case-control study	CLPTM1L	rs401681 C/T	C/T	55	200	255

48	Lu XZ, 2013	Lung cancer	Miscellany	China	Asian	Case-control study	CLPTM1L	rs402710 C/T	C/T	661	1062	1723
48	Lu XZ, 2013	Lung cancer	Miscellany	China	Asian	Case-control study	CLPTM1L	rs402710 C/T	C/T	421	1062	1483
49	Wu HJ, 2013	Lung cancer	Miscellany	China	Asian	Case-control study	TERT	rs2736098 C/T	C/T	539	627	1166
49	Wu HJ, 2013	Lung cancer	NSCLC (Adenocarcinoma)	China	Asian	Case-control study	TERT	rs2736098 C/T	C/T	293	627	920
49	Wu HJ, 2013	Lung cancer	NSCLC (Squamous cell carcinoma)	China	Asian	Case-control study	TERT	rs2736098 C/T	C/T	246	627	873
50	Jiang M, 2013	Lung cancer	Miscellany	China	Asian	Case-control study	CLPTM1L	rs401681 C/T	C/T	726	860	1586
50	Jiang M, 2013	Esophageal cancer	Squamous cell carcinoma	China	Asian	Case-control study	CLPTM1L	rs401681 C/T	C/T	753	860	1613
51	Wang FX, 2014	Lung cancer	Miscellany	China	Asian	Case-control study	TERT	rs2736100 C/A	C/A	1552	1605	3157
51	Wang FX, 2014	Lung cancer	NSCLC (Adenocarcinoma)	China	Asian	Case-control study	TERT	rs2736100 C/A	C/A	746	1605	2351
51	Wang FX, 2014	Lung cancer	NSCLC (Squamous cell carcinoma)	China	Asian	Case-control study	TERT	rs2736100 C/A	C/A	596	1605	2201
52	Liu CL, 2014	Lung cancer	Miscellany	China	Asian	Case-control study	TERT	rs2736098 C/T	C/T	980	1000	1980
52	Liu CL, 2014	Lung cancer	NSCLC (Adenocarcinoma)	China	Asian	Case-control study	TERT	rs2736098 C/T	C/T	504	1000	1504
52	Liu CL, 2014	Lung cancer	NSCLC (Squamous cell carcinoma)	China	Asian	Case-control study	TERT	rs2736098 C/T	C/T	220	1000	1220
52	Liu CL, 2014	Lung cancer	Miscellany	China	Asian	Case-control study	CLPTM1L	rs401681 C/T	C/T	980	1000	1980
52	Liu CL, 2014	Lung cancer	NSCLC (Adenocar	China	Asian	Case-control study	CLPTM1L	rs401681 C/T	C/T	504	1000	1504

			cinoma)									
52	Liu CL, 2014	Lung cancer	NSCLC (Squamous cell carcinoma)	China	Asian	Case-control study	CLPTM1L	rs401681 C/T	C/T	220	1000	1220
52	Liu CL, 2014	Lung cancer	Small cell carcinoma	China	Asian	Case-control study	CLPTM1L	rs401681 C/T	C/T	140	1000	1140
53	Zhang Y, 2014	Lung cancer	Miscellany	China	Asian	Case-control study	TERT	rs2736098 C/T	C/T	366	366	732
53	Zhang Y, 2014	Lung cancer	Miscellany	China	Asian	Case-control study	CLPTM1L	rs401681 C/T	C/T	366	366	732
54	Yin J, 2014	Esophageal cancer	Squamous cell carcinoma	China	Asian	Case-control study	CLPTM1L	rs401681 C/T	C/T	629	686	1315
55	Yin ZH, 2014	Lung cancer	Miscellany	China	Asian	Case-control study	TERT	rs2736100 C/A	C/A	524	524	1048
55	Yin ZH, 2014	Lung cancer	Miscellany	China	Asian	Case-control study	TERT/CLPTM1L	rs4975616 G/A	G/A	524	524	1048
56	Liang YQ, 2014	Lung cancer	Miscellany	China	Asian	Case-control study	TERT/CLPTM1L	rs4975616 G/A	G/A	309	310	619
56	Liang YQ, 2014	Lung cancer	Miscellany	China	Asian	Case-control study	CLPTM1L	rs402710 C/T	C/T	309	310	619
56	Liang YQ, 2014	Lung cancer	Miscellany	China	Asian	Case-control study	CLPTM1L	rs401681 C/T	C/T	309	310	619
57	Jager R, 2014	Myeloproliferative neoplasms (MPNs)	Miscellany	Italy	Caucasian	Case-control study	TERT	rs2736100 C/A	C/A	717	202	919
58	Singh V, 2014	Bladder cancer		India	Others	Cohort sytudy	TERT	rs2736098 C/T	C/T	225	240	465
59	Gao L, 2014	Lung cancer	Miscellany	China	Asian	Case-control study	TERT	rs2242652 G/A	G/A	309	310	619
59	Gao L, 2014	Lung cancer	Miscellany	China	Asian	Case-control study	TERT	rs2736098 C/T	C/T	309	310	619
59	Gao L, 2014	Lung cancer	Miscellany	China	Asian	Case-control study	TERT	rs4246742 T/A	T/A	309	310	619
60	Shadrina AS, 2015	Breast cancer		Russia	Caucasian	Case-control study	TERT	rs2853669 T/C	T/C	660	523	1183
60	Shadrina AS, 2015	Breast cancer		Russia	Caucasian	Case-control study	TERT	rs2853669 T/C	T/C	372	363	735
60	Shadrina AS, 2015	Breast cancer		Russia	Caucasian	Case-control study	TERT	rs2736100 C/A	C/A	660	523	1183

60	Shadrina AS, 2015	Breast cancer		Russia	Caucasian	Case-control study	TERT	rs2736100 C/A	C/A	372	363	735
61	Su LY, 2014	Hepatocellular carcinoma		China	Asian	Case-control study	TERT	rs2736098 C/T	C/T	201	210	411
62	Zhang Y, 2014	Bladder cancer		China	Asian	Case-control study	CLPTM1L	rs401681 C/T	C/T	367	420	787
63	Luo X, 2014	Lung cancer	Miscellany	China	Asian	Case-control study	CLPTM1L	rs31489 C/A	C/A	611	1062	1673
63	Luo X, 2014	Lung cancer	NSCLC	China	Asian	Case-control study	CLPTM1L	rs31489 C/A	C/A	427	1062	1489
64	Llorca-Cardeñosa MJ, 2014	Skin cancer	melanoma	Spain	Caucasian	Case-control study	CLPTM1L	rs401681 C/T	C/T	648	381	1029
64	Llorca-Cardeñosa MJ, 2014	Skin cancer	melanoma	Spain	Caucasian	Case-control study	CLPTM1L	rs401681 C/T	C/T	322	352	674
65	Hashemi M, 2014	Breast cancer		Iran	Caucasian	Case-control study	TERT	MNS16A L/S	L/S	266	225	491
65	Hashemi M, 2014	Breast cancer		Iran	Caucasian	Case-control study	TERT	rs2736098 C/T	C/T	266	225	491
65	Hashemi M, 2014	Breast cancer		Iran	Caucasian	Case-control study	TERT	rs2735940 A/G	A/G	266	225	491
66	Xun XJ, 2014	Lung cancer	Miscellany	China	Asian	Case-control study	CLPTM1L	rs401681 C/T	C/T	228	301	529
66	Xun XJ, 2014	Lung cancer	Miscellany	China	Asian	Case-control study	CLPTM1L	rs402710 C/T	C/T	228	301	529
67	Campa D, 2015	Pancreatic cancer		Italy, Germany, Czech Republic, Greece, Lithuania, Poland, United Kingdom	Caucasian	Case-control study	CLPTM1L	rs401681 C/T	C/T	1901	4106	6007
67	Campa D, 2015	Pancreatic cancer		Italy, Germany, Czech Republic,	Caucasian	Case-control study	TERT/CLPTM1L	rs4635969 G/A	G/A	1901	4106	6007

				Greece, Lithuania, Poland, United Kingdom								
68	Liu SG, 2015	Lung cancer	Miscellany	China	Asian	Case-control study	CLPTM1L	rs402710 C/T	C/T	304	319	623
68	Liu SG, 2015	Lung cancer	Miscellany	China	Asian	Case-control study	CLPTM1L	rs401681 C/T	C/T	304	319	623
68	Liu SG, 2015	Lung cancer	Miscellany	China	Asian	Case-control study	CLPTM1L	rs465498 A/G	A/G	304	319	623
68	Liu SG, 2015	Lung cancer	Miscellany	China	Asian	Case-control study	TERT	rs2736100 C/A	C/A	304	319	623
69	Fahmideh MA, 2015	Glioma		Sweden, Denmark, Norway and Switzerland	Caucasian	Case-control study	TERT	rs2736100 C/A	C/A	245	489	534
70	Du JB, 2015	Gastric cancer		China	Asian	Case-control study	TERT	rs2736100 C/A	C/A	1136	1012	2148
71	Wei RR, 2015	Lung cancer	NSCLC	China	Asian	Case-control study	TERT	rs2736100 C/A	C/A	714	2520	3234
71	Wei RR, 2015	Lung cancer	NSCLC	China	Asian	Case-control study	TERT	rs2736100 C/A	C/A	296	2520	2816
71	Wei RR, 2015	Lung cancer	NSCLC	China	Asian	Case-control study	TERT	rs2736100 C/A	C/A	406	2520	2926
72	Wang JQ, 2016	Esophageal cancer	Squamous cell carcinoma	China	Asian	Case-control study	CLPTM1L	rs401681 C/T	C/T	360	310	670
73	Choi BJ, 2015	Gastric cancer		Korea	Asian	Case-control study	TERT	rs2736100 C/A	C/A	243	246	489
74	Weissfeld JL, 2015	Lung cancer	Miscellany	USA	Caucasian	Case-control study	TERT	rs2736100 C/A	C/A	778	1166	1944
75	Yoo SS, 2015	Lung cancer	Miscellany	Korea	Asian	Case-control study	TERT	rs2736098 C/T	C/T	1100	1096	2196
76	Krahling T, 2016	Myeloproliferative neoplasms (MPNs)	Miscellany	Hungary	Caucasian	Case-control study	TERT	rs2736100 C/A	C/A	584	400	984
76	Krahling T, 2016	Myeloprolif	Polycythe	Hungary	Caucasian	Case-control study	TERT	rs2736100 C/A	C/A	209	400	609

		erative neoplasms (MPNs)	mia vera (PV)									
76	Krahling T, 2016	Myeloproliferative neoplasms (MPNs)	Essential thrombocythemia (ET)	Hungary	Caucasian	Case-control study	TERT	rs2736100 C/A	C/A	281	400	609
76	Krahling T, 2016	Myeloproliferative neoplasms (MPNs)	Primary myelofibrosis (PMF)	Hungary	Caucasian	Case-control study	TERT	rs2736100 C/A	C/A	94	400	494
77	Jannuzzi AT, 2015	Colorectal cancer	Miscellany	Turkey	Caucasian	Case-control study	TERT	rs2736100 C/A	C/A	104	135	239
78	Zhou LQ, 2016	Esophageal cancer	Squamous cell carcinoma	China	Asian	Case-control study	TERT	rs2853691 A/G	A/G	588	600	1188
78	Zhou LQ, 2016	Esophageal cancer	Squamous cell carcinoma	China	Asian	Case-control study	TERT	rs2736100 C/A	C/A	588	600	1188
78	Zhou LQ, 2016	Esophageal cancer	Squamous cell carcinoma	China	Asian	Case-control study	CLPTM1L	rs451360 C/A	C/A	588	600	1188
78	Zhou LQ, 2016	Esophageal cancer	Squamous cell carcinoma	China	Asian	Case-control study	TERT	rs2853691 A/G	A/G	1000	1000	2000
78	Zhou LQ, 2016	Esophageal cancer	Squamous cell carcinoma	China	Asian	Case-control study	TERT	rs2853691 A/G	A/G	510	550	1060
78	Zhou LQ, 2016	Esophageal cancer	Squamous cell carcinoma	China	Asian	Case-control study	TERT	rs2736100 C/A	C/A	1000	1000	2000
78	Zhou LQ, 2016	Esophageal cancer	Squamous cell	China	Asian	Case-control study	TERT	rs2736100 C/A	C/A	510	550	1060

			carcinoma									
78	Zhou LQ, 2016	Esophageal cancer	Squamous cell carcinoma	China	Asian	Case-control study	CLPTM1L	rs451360 C/A	C/A	1000	1000	2000
78	Zhou LQ, 2016	Esophageal cancer	Squamous cell carcinoma	China	Asian	Case-control study	CLPTM1L	rs451360 C/A	C/A	510	550	1060
79	Jin TB, 2016	Lung cancer	Miscellany	China	Asian	Case-control study	CLPTM1L	rs402710 C/T	C/T	554	696	1250
79	Jin TB, 2016	Lung cancer	Miscellany	China	Asian	Case-control study	CLPTM1L	rs401681 C/T	C/T	554	696	1250
80	Bayram S, 2016	Gastric cancer		Turkey	Caucasian	Case-control study	TERT	rs2735940 A/G	A/G	104	209	313
80	Bayram S, 2016	Gastric cancer		Turkey	Caucasian	Case-control study	TERT	rs2736100 C/A	C/A	104	209	313
81	Trifa AP, 2016	Myeloproliferative neoplasms (MPNs)	Miscellany	multiple countries	Caucasian	Case-control study	TERT	rs2736100 C/A	C/A	529	433	962
81	Trifa AP, 2016	Myeloproliferative neoplasms (MPNs)	Polycythemia vera (PV)	multiple countries	Caucasian	Case-control study	TERT	rs2736100 C/A	C/A	184	433	617
81	Trifa AP, 2016	Myeloproliferative neoplasms (MPNs)	Essential thrombocythemia (ET)	multiple countries	Caucasian	Case-control study	TERT	rs2736100 C/A	C/A	271	433	704
81	Trifa AP, 2016	Myeloproliferative neoplasms (MPNs)	Primary myelofibrosis (PMF)	multiple countries	Caucasian	Case-control study	TERT	rs2736100 C/A	C/A	74	433	507
82	Xing YL,2016	Lung cancer	NSCLC	China	Asian	Case-control study	TERT	rs2736100 C/A	C/A	418	410	828
82	Xing YL, 2016	Lung cancer	NSCLC	China	Asian	Case-control study	TERT	rs2853677 G/A	G/A	418	410	828
83	Wang X, 2016	Lung cancer	Miscellany	China	Asian	Case-control study	TERT	rs2736100 C/A	C/A	500	500	1000
84	Ge MH, 2016	Thyroid	papillary	China	Asian	Case-control study	TERT	rs2736100 C/A	C/A	500	500	1000

		cancer	thyroid carcinoma									
84	Ge MH, 2016	Thyroid cancer	papillary thyroid carcinoma	China	Asian	Case-control study	TERT	rs2736100 C/A	C/A	1000	1000	2000
84	Ge MH, 2016	Thyroid cancer	papillary thyroid carcinoma	China	Asian	Case-control study	TERT	rs2736100 C/A	C/A	800	800	1600
85	Li XT,2016	Lung cancer	Miscellany	China	Asian	Case-control study	TERT	rs2853677 G/A	G/A	391	337	728
85	Li XT,2016	Lung cancer	Miscellany	China	Asian	Case-control study	TERT	rs2736100 C/A	C/A	391	337	728
86	Oztas E, 2016	Breast cancer		Turkey	Caucasian	Case-control study	TERT	rs2853669 T/C	T/C	107	110	217
86	Oztas E, 2016	Breast cancer		Turkey	Caucasian	Case-control study	TERT	rs2736100 C/A	C/A	107	110	217
86	Oztas E, 2016	Breast cancer		Turkey	Caucasian	Case-control study	TERT	rs2736098 C/T	C/T	107	110	217
87	Gong L, 2016	Thyroid cancer		China	Asian	Case-control study	TERT	rs2736100 C/A	C/A	452	452	904
88	Shiraishi K, 2016	Lung cancer	NSCLC (Adenocarcinoma)	Japan	Asian	GWAS	TERT	rs2736100 C/A	C/A	663	4367	5030
88	Shiraishi K, 2016	Lung cancer	NSCLC (Adenocarcinoma)	Japan	Asian	Case-control study	TERT	rs2736100 C/A	C/A	1748	6817	8565
88	Shiraishi K, 2016	Lung cancer	NSCLC (Adenocarcinoma)	Japan	Asian	Case-control study	TERT	rs2736100 C/A	C/A	1315	3974	5289
88	Shiraishi K, 2016	Lung cancer	NSCLC (Adenocarcinoma)	Japan	Asian	GWAS	TERT	rs2853677 G/A	G/A	663	4367	5030
88	Shiraishi K, 2016	Lung cancer	NSCLC (Adenocarcinoma)	Japan	Asian	Case-control study	TERT	rs2853677 G/A	G/A	1748	6817	8565

88	Shiraishi K, 2016	Lung cancer	NSCLC (Adenocarcinoma)	Japan	Asian	Case-control study	TERT	rs2853677 G/A	G/A	1315	3974	5289
89	Dahlström J, 2016	Myeloproliferative neoplasms (MPNs)	Miscellany	China	Asian	Case-control study	TERT	rs2736100 C/A	C/A	101	101	202
89	Dahlström J, 2016	Myeloproliferative neoplasms (MPNs)	Miscellany	Sweden	Caucasian	Case-control study	TERT	rs2736100 C/A	C/A	126	756	882
90	Duan XL, 2016	Gastric cancer		China	Asian	Case-control study	TERT	rs10069690 C/T	C/T	302	300	605
90	Duan XL, 2016	Gastric cancer		China	Asian	Case-control study	TERT	rs2853676 T/C	T/C	302	300	605
91	Dong J, 2017	Lung cancer	NSCLC	China	Asian	Case-control study	TERT	rs2736100 C/A	C/A	200	300	500
91	Dong J, 2017	Lung cancer	NSCLC	China	Asian	Case-control study	TERT	rs2853677 G/A	G/A	200	300	500
91	Dong J, 2017	Lung cancer	NSCLC	China	Asian	Case-control study	CLPTM1L	rs401681 C/T	C/T	200	300	500
92	Yuan XT, 2017	Hepatocellular carcinoma		China	Asian	Case-control study	TERT	rs2736098 C/T	C/T	245	257	502
93	Zhang J, 2017	Gastric cancer		China	Asian	Case-control study	TERT	rs2736100 C/A	C/A	360	728	1088
93	Zhang J, 2017	Gastric cancer		China	Asian	Case-control study	TERT	rs2735940 A/G	A/G	360	728	1088
94	Xiao XY, 2017	Lung cancer	Miscellany	China	Asian	Case-control study	TERT	rs2736098 C/T	C/T	214	216	430
94	Xiao XY, 2017	Lung cancer	Miscellany	China	Asian	Case-control study	CLPTM1L	rs401681 C/T	C/T	214	216	430
95	Chiang YH, 2017	Myeloproliferative neoplasms (MPNs)	Miscellany	China	Asian	Case-control study	TERT	rs2736100 C/A	C/A	178	16946	17124
95	Chiang YH, 2017	Myeloproliferative neoplasms	Polycythemia vera (PV)	China	Asian	Case-control study	TERT	rs2736100 C/A	C/A	54	16946	17000

		(MPNs)										
95	Chiang YH, 2017	Myeloproliferative neoplasms (MPNs)	Essential thrombocythemia (ET)	China	Asian	Case-control study	TERT	rs2736100 C/A	C/A	109	16946	17055
95	Chiang YH, 2017	Myeloproliferative neoplasms (MPNs)	Primary myelofibrosis (PMF)	China	Asian	Case-control study	TERT	rs2736100 C/A	C/A	15	16946	16961
96	Ye G, 2017	Lung cancer	Miscellany	China	Asian	Case-control study	TERT	rs2242652 G/A	G/A	554	603	1157
96	Ye G, 2017	Lung cancer	Miscellany	China	Asian	Case-control study	TERT	rs2853677 G/A	G/A	554	603	1157
97	Aydin M, 2018	Breast cancer		Turkey	Caucasian	Case-control study	TERT	rs2736109 G/A	G/A	123	122	245
97	Aydin M, 2018	Breast cancer		Turkey	Caucasian	Case-control study	TERT	rs2735940 A/G	A/G	123	122	245
97	Aydin M, 2018	Breast cancer		Turkey	Caucasian	Case-control study	TERT	rs2853669 T/C	T/C	123	122	245
97	Aydin M, 2018	Breast cancer		Turkey	Caucasian	Case-control study	TERT	rs2736098 C/T	C/T	123	122	245
97	Aydin M, 2018	Breast cancer		Turkey	Caucasian	Case-control study	TERT	rs2736100 C/A	C/A	123	122	245
98	Li CY, 2018	Lung cancer	NSCLC	China	Asian	Case-control study	TERT	rs2853677 G/A	G/A	467	526	993
99	Bagherpour M, 2018	Breast cancer		Iran	Caucasian	Case-control study	TERT	rs2736098 C/T	C/T	105	110	215
100	Li Y, 2019	Lung cancer	NSCLC (Adenocarcinoma)	China	Asian	Case-control study	TERT	rs2853677 G/A	G/A	605	970	1575
101	Zhang NS, 2019	Gastric cancer	Gastric cardia adenocarcinoma (GCA)	China	Asian	Case-control study	TERT	rs10069690 C/T	C/T	584	568	1152
101	Zhang NS, 2019	Gastric	Gastric	China	Asian	Case-control study	TERT	rs10069690	C/T	584	568	1152

		cancer	cardia adenocarcinoma (GCA)					C/T				
101	Zhang NS, 2019	Gastric cancer	Gastric cardia adenocarcinoma (GCA)	China	Asian	Case-control study	TERT	rs2853676 T/C	T/C	440	500	940
101	Zhang NS, 2019	Gastric cancer	Gastric cardia adenocarcinoma (GCA)	China	Asian	Case-control study	TERT	rs2853676 T/C	T/C	440	500	940
102	Rasool Bhat Gh, 2019	Lung cancer	Miscellany	India	Others	Case-control study	TERT	rs2853677 G/A	G/A	178	400	578
102	Rasool Bhat Gh, 2019	Lung cancer	NSCLC (Adenocarcinoma)	India	Others	Case-control study	TERT	rs2853677 G/A	G/A	112	400	512
103	Matsuguma M, 2019	Myeloproliferative neoplasms (MPNs)	Miscellany	Japan	Asian	Case-control study	TERT	rs2736100 C/A	C/A	201	366	567
103	Matsuguma M, 2019	Myeloproliferative neoplasms (MPNs)	Polycythemia vera (PV)	Japan	Asian	Case-control study	TERT	rs2736100 C/A	C/A	52	366	418
103	Matsuguma M, 2019	Myeloproliferative neoplasms (MPNs)	Essential thrombocythemia (ET)	Japan	Asian	Case-control study	TERT	rs2736100 C/A	C/A	131	366	497
103	Matsuguma M, 2019	Myeloproliferative	Primary myelofibro	Japan	Asian	Case-control study	TERT	rs2736100 C/A	C/A	18	366	384

		neoplasms (MPNs)	sis (PMF)									
104	Yoo SS, 2020	Lung cancer	Miscellany	Korea	Asian	Case-control study	TERT/CLPTM1L	rs4975616 G/A	G/A	714	626	1340
105	Arimura-Omori M, 2020	Idiopathic pulmonary fibrosis (IPF)		Japan	Asian	Case-control study	TERT	rs2736100 C/A	C/A	155	379	534
106	Ma LL, 2020	Gastric cancer		China	Asian	Case-control study	TERT	rs10069690 C/T	C/T	1000	1100	2100
106	Ma LL, 2020	Gastric cancer		China	Asian	Case-control study	TERT	rs2853676 T/C	T/C	1000	1100	2100
107	Pandith AA, 2020	Glioma		India	Others	Case-control study	TERT	rs2736100 C/A	C/A	106	210	316
108	Mandour I, 2020	Lung cancer	Miscellany	Egypt	African	Case-control study	TERT	rs2736100 C/A	C/A	40	40	80
109	Wang HJ, 2016	Lung cancer	Miscellany	China	Asian	Case-control study	TERT	rs4246742 T/A	T/A	228	310	538
109	Wang HJ, 2016	Lung cancer	Miscellany	China	Asian	Case-control study	TERT	rs2736098 C/T	C/T	228	310	538

Note: A, adenine; C, cytosine; G, guanine; T, thymine; GWAS: genome-wide association studies; NSCLC: non-small cell lung cancer.

References:

1. Savage SA, Chanock SJ, Lissowska J, Brinton LA, Richesson D, Peplonska B, et al. Genetic variation in five genes important in telomere biology and risk for breast cancer. Br J Cancer. 2007 Sep 17;97(6):832-6.
2. Wang Y, Hu ZB, Liang J, Wang ZW, Tang JH, Wang S, et al. A tandem repeat of human telomerase reverse transcriptase (hTERT) and risk of breast cancer development and metastasis in Chinese women. Carcinogenesis. 2008 Jun;29(6):1197-201.
3. Mushiroda T, Wattanapokayakit S, Takahashi A, Nukiwa T, Kudoh S, Ogura T, et al. A genome-wide association study identifies an association of a common variant in TERT with susceptibility to idiopathic pulmonary fibrosis. J Med Genet. 2008 Oct;45(10):654-6.
4. Wang YF, Broderick P, Webb E, Wu XF, Vijayakrishnan J, Matakidou A, et al. Common 5p15.33 and 6p21.33 variants influence lung cancer risk. Nat Genet. 2008 Dec;40(12):1407-9.
5. Jin GF, Xu L, Shu YQ, Tian T, Liang J, Xu Y, et al. Common genetic variants on 5p15.33 contribute to risk of lung adenocarcinoma in a Chinese population. Carcinogenesis. 2009 Jun;30(6):987-90.
6. Varadi V, Brendle A, Grzybowska E, Johansson R, Enquist E, Butkiewicz D, et al. A functional promoter polymorphism in the TERT gene does not affect inherited susceptibility to breast cancer. Cancer Genet Cytogenet. 2009 Apr 15;190(2):71-4.
7. Zienolddiny S, Skaug V, Landvik NE, Ryberg D, Phillips DH, Houlston R, et al. The TERT-CLPTM1L lung cancer susceptibility variant associates with higher DNA adduct formation in the lung. Carcinogenesis. 2009 Aug;30(8):1368-71.
8. Shete S, Hosking FJ, Robertson LB, Dobbins SE, Sanson M, Malmer B, et al. Genome-wide association study identifies five susceptibility loci for glioma. Nat Genet. 2009 Aug;41(8):899-904.
9. Broderick P, Wang YF, Vijayakrishnan J, Matakidou A, Spitz MR, Eisen T, et al. Deciphering the impact of common genetic variation on lung cancer risk: a genome-wide association study. Cancer Res. 2009 Aug 15;69(16):6633-41.
10. Varadi V, Brendle A, Brandt A, Johansson R, Enquist K, Henriksson R, et al. Polymorphisms in telomere-associated genes, breast cancer susceptibility and prognosis. Eur J Cancer. 2009 Nov;45(17):3008-16.
11. Choi JE, Kang HG, Jang JS, Choi YY, Kim MJ, Kim JS, et al. Polymorphisms in telomere maintenance genes and risk of lung cancer. Cancer Epidemiol Biomarkers Prev. 2009 Oct;18(10):2773-81.
12. Landi MT, Chatterjee N, Yu K, Goldin LR, Goldstein AM, Rotunno M, et al. A genome-wide association study of lung cancer identifies a region of chromosome 5p15 associated with risk for adenocarcinoma. Am J Hum Genet. 2009 Nov;85(5):679-91.
13. Wang YF, Broderick P, Matakidou A, Eisen T, Houlston TS. Role of 5p15.33 (TERT-CLPTM1L), 6p21.33 and 15q25.1 (CHRNA5-CHRNA3) variation and lung cancer risk in never-smokers. Carcinogenesis. 2010 Feb;31(2):234-8.
14. Shen J, Gammon MD, Wu HC, Terry MB, Wang Q, Bradshaw PT, et al. Multiple genetic variants in telomere pathway genes and breast cancer risk. Cancer Epidemiol Biomarkers Prev. 2010 Jan; 19(1):219-28.
15. Truong T, Hung RJ, Amos CI, Wu XF, Bickeböller H, Rosenberger A, et al. Replication of lung cancer susceptibility loci at chromosomes 15q25, 5p15, and 6p21: a pooled analysis from the International Lung Cancer Consortium. J Natl Cancer Inst. 2010 Jul 7;102(13):959-71.
16. Hsiung CA, Lan Q, Hong YC, Chen CJ, Hosgood HD, Chang IS, et al. The 5p15.33 locus is associated with risk of lung adenocarcinoma in never-smoking females in Asia. PLoS Genet. 2010 Aug 5;6(8):e1001051.
17. Miki D, Kubo M, Takahashi A, Yoon KA, Kim J, Lee KL, et al. Variation in TP63 is associated with lung adenocarcinoma susceptibility in Japanese and Korean populations. Nat Genet. 2010 Oct;42(10):893-6.
18. Yoon KA, Park JH, Han J, Park S, Lee GK, Han JY, et al. A genome-wide association study reveals susceptibility variants for non-small cell lung cancer in the Korean population. Hum Mol Genet. 2010 Dec 15;19(24):4948-54.
19. Manuela Gago-Dominguez 1, Xuejuan Jiang, David V Conti, Jose Esteban Castelao, Mariana C Stern, Victoria K Cortessis, et al. Genetic variations on chromosomes 5p15 and 15q25 and bladder cancer risk: findings from the Los Angeles-Shanghai bladder case-control study. Carcinogenesis. 2011 Feb;32(2):197-202.
20. Nan HM, Qureshi AA, Prescott J, Vivo ID, Han JL, et al. Genetic variants in telomere-maintaining genes and skin cancer risk. Hum Genet. 2011 Mar;129(3):247-53.

21. Chen HY, Chen YY, Zhao Y, Fan WW, Zhou KK, Liu YH, et al. Association of sequence variants on chromosomes 20, 11, and 5 (20q13.33, 11q23.3, and 5p15.33) with glioma susceptibility in a Chinese population. Am J Epidemiol. 2011 Apr 15;173(8):915-22.
22. Kohno T, Kunitoh H, Mimaki S, Shiraishi K, Kuchiba A, Yamamoto S, et al. Contribution of the TP53, OGG1, CHRNA3, and HLA-DQA1 genes to the risk for lung squamous cell carcinoma. J Thorac Oncol. 2011 Apr;6(4):813-7.
23. Hu Z, Wu C, Shi YY, Guo H, Zhao XY, Yin ZH, et al. A genome-wide association study identifies two new lung cancer susceptibility loci at 13q12.12 and 22q12.2 in Han Chinese. Nat Genet. 2011 Jul 3;43(8):792-6.
24. Pande M, Spitz MR, Wu XF, Gorlov IP, Chen WV, Amos CI, et al. Novel genetic variants in the chromosome 5p15.33 region associate with lung cancer risk. Carcinogenesis. 2011 Oct;32(10):1493-9.
25. Beesley J, Pickett HA, Johnatty SE, Dunning AM, Chen XQ, Jun Li, et al. Functional polymorphisms in the TERT promoter are associated with risk of serous epithelial ovarian and breast cancers. PLoS One. 2011;6(9):e24987.
26. Jaworowska E, Trubicka J, Lener MR, Masojć B, Złowocka-Perłowska E, McKay JD, et al. Smoking related cancers and loci at chromosomes 15q25, 5p15, 6p22.1 and 6p21.33 in the Polish population. PLoS One. 2011;6(9):e25057.
27. Rizzato C, Campa D, Giese N, Werner J, Rachakonda PS, Kumar R, et al. Pancreatic cancer susceptibility loci and their role in survival. PLoS One. 2011;6(11):e27921.
28. Pellatt AJ, Wolff RK, Herrick J, Lundgreen A, Slattery ML. TERT's role in colorectal carcinogenesis. Mol Carcinog. 2013 Jul;52(7):507-13.
29. Chen XF, Cai S, Chen QG, Ni ZH, Tang JH, Xu DW, et al. Multiple variants of TERT and CLPTM1L constitute risk factors for lung adenocarcinoma. Genet Mol Res. 2012 Feb 16;11(1):370-8.
30. Bae EY, Lee SY, Kang BK, Lee EJ, Choi YY, Kang HG, et al. Replication of results of genome-wide association studies on lung cancer susceptibility loci in a Korean population. Respirology. 2012 May;17(4):699-706.
31. Ito H, McKay JD, Hosono S, Hida T, Yatabe Y, Mitsudomi T, et al. Association between a genome-wide association study-identified locus and the risk of lung cancer in Japanese population. J Thorac Oncol. 2012 May;7(5):790-8.
32. Hofer P, Baierl A, Bernhart K, Leeb G, Mach K, Micksche M, et al. Association of genetic variants of human telomerase with colorectal polyps and colorectal cancer risk. Mol Carcinog. 2012 Oct;51 Suppl 1:E176-82.
33. Ma ZC, Hu QF, Chen Z, Tao S, Macnamara L, Kim ST, et al. Systematic evaluation of bladder cancer risk-associated single-nucleotide polymorphisms in a Chinese population. Mol Carcinog. 2013 Nov;52(11):916-21.
34. Kinnersley B, Migliorini G, Broderick P, Whiffin N, Dobbins SE, Casey G, et al. The TERT variant rs2736100 is associated with colorectal cancer risk. Br J Cancer. 2012 Sep 4;107(6):1001-8.
35. Zagouri F, Sergentanis TN, Gazouli M, Tsigginou A, Dimitrakakis C, Eleutherakis-Papaiakovou E, et al. HTERT MNS16A polymorphism in breast cancer: a case-control study. Mol Biol Rep. 2012 Dec;39(12):10859-63.
36. Wang H, Zhao Y, Ma J, Zhang G, Mu Y, Qi G, et al. The genetic variant rs401681C/T is associated with the risk of non-small cell lung cancer in a Chinese mainland population. Genet Mol Res. 2013 Jan 22;12(1):67-73.
37. Zhao ZH, Li C, Yang LX, Zhang XB, Zhao XY, Song X, et al. Significant association of 5p15.33 (TERT-CLPTM1L genes) with lung cancer in Chinese Han population. Exp Lung Res. 2013 Mar;39(2):91-8.
38. Zhang C, Tian YP, Wang Y, Guo FH, Qin JF, Ni H. hTERT rs2736098 genetic variants and susceptibility of hepatocellular carcinoma in the Chinese population: a case-control study. Hepatobiliary Pancreat Dis Int. 2013 Feb;12(1):74-9.
39. Myneni AA, Chang SC, Niu RH, Liu L, Ochs-Balcom HM, Li Y, et al. Genetic polymorphisms of TERT and CLPTM1L and risk of lung cancer--a case-control study in a Chinese population. Lung Cancer. 2013 May;80(2):131-7.

40. Lan Q, Cawthon R, Gao YT, Hu W, Hosgood 3rd HD, Barone-Adesi F, et al. Longer telomere length in peripheral white blood cells is associated with risk of lung cancer and the rs2736100 (CLPTM1L-TERT) polymorphism in a prospective cohort study among women in China. PLoS One. 2013;8(3):e59230.
41. Pellatt AJ, Wolff RK, Torres-Mejia G, John EM, Herrick JS, Lundgreen A, et al. Telomere length, telomere-related genes, and breast cancer risk: the breast cancer health disparities study. Genes Chromosomes Cancer. 2013 Jul;52(7):595-609.
42. Ke JT, Zhong R, Zhang T, Liu LF, Rui R, Shen N, et al. Replication study in Chinese population and meta-analysis supports association of the 5p15.33 locus with lung cancer. PLoS One. 2013 Apr 30;8(4):e62485.
43. Li C, Yin YH, Wu W, Li XL, Ren YW, Zhou B. Genetic variations in TERT-CLPTM1L genes and risk of lung cancer in Chinese women nonsmokers.PLoS One. 2013 May 30;8(5):e64988.
44. Iizuka T, Sawabe T, Takubo K, Liu M, Homma Y, Suzuki M, et al. hTERT promoter polymorphism, -1327C>T, is associated with the risk of epithelial cancer. Springerplus. 2013 May 31;2(1):249.
45. Jin TB, Zhang JY, Li G, Du SL, Geng TT, Gao J, et al. RTEL1 and TERT polymorphisms are associated with astrocytoma risk in the Chinese Han population. Tumour Biol. 2013 Dec;34(6):3659-66.
46. de Mello RA, Ferreira M, Soares-Pires F, Costa S, Cunha J, Oliveira P, et al. The impact of polymorphic variations in the 5p15, 6p12, 6p21 and 15q25 Loci on the risk and prognosis of portuguese patients with non-small cell lung cancer. PLoS One. 2013 Sep 6;8(9):e72373.
47. Sun Y, Zhang YJ, Kong XM. No association of XRCC1 and CLPTM1L polymorphisms with non-small cell lung cancer in a non-smoking Han Chinese population. Asian Pac J Cancer Prev. 2013;14(9):5171-4.
48. Lu XZ, Ke JT, Luo X, Zhu YW, Zou L, Li HJ, et al. The SNP rs402710 in 5p15.33 is associated with lung cancer risk: a replication study in Chinese population and a meta-analysis. PLoS One. 2013 Oct 23;8(10):e76252.
49. Wu HJ, Qiao NA, Wang Y, Jiang M, Wang SK, Wang, CH et al. Association between the telomerase reverse transcriptase (TERT) rs2736098 polymorphism and cancer risk: evidence from a case-control study of non-small-cell lung cancer and a meta-analysis. PLoS One. 2013 Nov 19;8(11):e76372.
50. Jiang M, Wu HJ, Qin CY. Genetic variant rs401681 at 5p15.33 modifies susceptibility to lung cancer but not esophageal squamous cell carcinoma. PLoS One. 2013 Dec 30;8(12):e84277.
51. Wang FX, Fu P, Pang YX, Liu CX, Shao ZL, Zhu JY, et al. TERT rs2736100T/G polymorphism upregulates interleukin 6 expression in non-small cell lung cancer especially in adenocarcinoma. Tumour Biol. 2014 May;35(5):4667-72.
52. Liu CL, Wang YJ, Huang H, Wang C, Zhang H, Kong YL, et al. Association between CLPTM1L-TERT rs401681 polymorphism and pancreatic cancer risk among Chinese Han population. Tumour Biol. 2014 Jun;35(6):5453-7.
53. Zhang Y, Zhao MM, Shen L, Ren YW, Su LY, Li XL, et al. Genetic polymorphisms of TERT and CLPTM1L and risk of lung cancer: a case-control study in northeast Chinese male population. Med Oncol. 2014 Jul;31(7):18.
54. Yin J, Wang LM, Zheng L, Wang X, Shi YJ, Shao AZ, et al. TERT-CLPTM1L Rs401681 C>T polymorphism was associated with a decreased risk of esophageal cancer in a Chinese population. PLoS One. 2014 Jul 9;9(7):e100667.
55. Yin ZH, Cui ZG, Ren YW, Zhang HB, Yan Y, Zhao YX, et al. Genetic polymorphisms of TERT and CLPTM1L, cooking oil fume exposure, and risk of lung cancer: a case-control study in a Chinese non-smoking female population. Med Oncol. 2014 Aug;31(8):114.
56. Liang YQ, Thakur A, Gao L, Wang T, Zhang S, Ren H, et al. Correlation of CLPTM1L polymorphisms with lung cancer susceptibility and response to cisplatin-based chemotherapy in a Chinese Han population. Tumour Biol. 2014 Dec;35(12):12075-82.
57. Jäger R, Harutyunyan AS, Rumi E, Pietra D, Berg T, Olcaydu D, et al. Common germline variation at the TERT locus contributes to familial clustering of myeloproliferative neoplasms. Am J Hematol. 2014 Dec;89(12):1107-10.
58. Singh V, Jaiswal PK, Mittal RD. Replicative study of GWAS TP63C/T, TERTC/T, and SLC14A1C/T with susceptibility to bladder cancer in North Indians. Urol Oncol. 2014 Nov;32(8):1209-14.

59. Gao L, Thakur A, Liang YQ, Zhang S, Wang T, Chen TJ, et al. Polymorphisms in the TERT gene are associated with lung cancer risk in the Chinese Han population. Eur J Cancer Prev. 2014 Nov;23(6):497-501.
60. Shadrina AS, Boyarskikh UA, Oskina NA, Sinkina TV, Lazarev AF, Petrova VD, et al. TERT polymorphisms rs2853669 and rs7726159 influence on prostate cancer risk in Russian population. Tumour Biol. 2015 Feb;36(2):841-7.
61. Su LY, Li XL, Shen L, Zhang Y, Zhao MM, Yin ZH, et al. Polymorphisms of TERT and CLPTM1L and the risk of hepatocellular carcinoma in Chinese males. Asian Pac J Cancer Prev. 2014;15(19):8197-201
62. Zhang Y, Sun Y, Chen T, Hu HL, Xie WQ, Qiao ZH, et al. Genetic variations rs11892031 and rs401681 are associated with bladder cancer risk in a Chinese population. Int J Mol Sci. 2014 Oct 24;15(11):19330-41.
63. Luo X, Lamsal LP, Xu WJ, Lu J, Lu YJ, Shen Y, et al. Genetic variant in CLPTM1L confers reduced risk of lung cancer: a replication study in Chinese and a meta-analysis. Asian Pac J Cancer Prev. 2014;15(21):9241-7.
64. Llorca-Cardeñosa MJ, Peña-Chilet M, Mayor M, Gomez-Fernandez G, Casado B, Martin-Gonzalez M, et al. Long telomere length and a TERT-CLPTM1 locus polymorphism association with melanoma risk. Eur J Cancer. 2014 Dec;50(18):3168-77.
65. Hashemi M, Amininia S, Ebrahimi M, Hashemi SM, Taheri M, Ghavami S. Association between hTERT polymorphisms and the risk of breast cancer in a sample of Southeast Iranian population. BMC Res Notes. 2014 Dec 10;7:895.
66. Xun XJ, Wang HJ, Yang H, Wang H, Wang B, Kang LL, et al. CLPTM1L genetic polymorphisms and interaction with smoking and alcohol drinking in lung cancer risk: a case-control study in the Han population from northwest China. Medicine (Baltimore). 2014 Dec;93(28):e289.
67. Campa D, Rizzato C, Stolzenberg-Solomon R, Pacetti P, Vodicka P, Cleary SP, et al. TERT gene harbors multiple variants associated with pancreatic cancer susceptibility. Int J Cancer. 2015 Nov 1;137(9):2175-83.
68. Liu SG, Ma L, Cen QH, Huang JS, Zhang JX, Zhang JJ. Association of genetic polymorphisms in TERT-CLPTM1L with lung cancer in a Chinese population. Genet Mol Res. 2015 May 4;14(2):4469-76.
69. Fahmideh MA, Lavebratt C, Schüz J, Röösli M, Tynes T, Grotzer MA, et al. CCDC26, CDKN2BAS, RTEL1 and TERT Polymorphisms in pediatric brain tumor susceptibility. Carcinogenesis. 2015 Aug;36(8):876-82.
70. Du JB, Zhu X, Xie CW, Dai NB, Gu YY, Zhu M, et al. Telomere length, genetic variants and gastric cancer risk in a Chinese population. Carcinogenesis. 2015 Sep;36(9):963-70.
71. Wei RR, Cao L, Pu HY, Wang HW, Zheng YL, Niu XM, et al. TERT Polymorphism rs2736100-C Is Associated with EGFR Mutation-Positive Non-Small Cell Lung Cancer. Clin Cancer Res. 2015 Nov 15;21(22):5173-5180.
72. Wang JQ, Zhang BP, Yang Z, Zhou L, Geng TT, Li HP, et al. Association of gastrointestinal gland cancer susceptibility loci with esophageal carcinoma among the Chinese Han population: a case-control study. Tumour Biol. 2016 Feb;37(2):1627-33.
73. Choi BJ, Yoon JH, Kim O, Choi WS, Nam SW, Lee JY, et al. Influence of the hTERT rs2736100 polymorphism on telomere length in gastric cancer. World J Gastroenterol. 2015 Aug 21;21(31):9328-36.
74. Weissfeld JL, Lin Y, Lin HM, Kurland BF, Wilson DO, Fuhrman CR, et al. Lung Cancer Risk Prediction Using Common SNPs Located in GWAS-Identified Susceptibility Regions. J Thorac Oncol. 2015 Nov;10(11):1538-45.
75. Yoo SS, Do SK, Choi JE, Lee SY, Lee J, Cha SI, et al. TERT Polymorphism rs2853669 Influences on Lung Cancer Risk in the Korean Population. J Korean Med Sci. 2015 Oct;30(10):1423-8.
76. Krahling T, Balassa K, Kiss KP, Bors A, Batai A, Halm G, et al. Co-occurrence of Myeloproliferative Neoplasms and Solid Tumors Is Attributed to a Synergism Between Cytoreductive Therapy and the Common TERT Polymorphism rs2736100. Cancer Epidemiol Biomarkers Prev. 2016 Jan;25(1):98-104.
77. Jannuzzi AT, Karaman E, Oztas E, Yanar HT, Özhan G. Telomerase Reverse Transcriptase (TERT) Gene Variations and Susceptibility of Colorectal Cancer. Genet Test Mol Biomarkers. 2015 Dec;19(12):692-7.
78. Zhou LQ, Fu GB, Wei JY, Shi J, Pan WT, Ren YL, et al. The identification of two regulatory ESCC susceptibility genetic variants in the TERT-CLPTM1L loci. Oncotarget. 2016 Feb 2;7(5):5495-506.

79. Jin TB, Li B, He N, Zhang Y, Xia RD, Kang LI, et al. CLPTM1L polymorphism as a protective factor for lung cancer: a case-control study in southern Chinese population. Tumour Biol. 2016 Aug;37(8):10533-8.
80. Bayram S, Ülger Y, Sümbül AT, Kaya BY, Genç A, Rencüzoğullari E, et al. Polymorphisms in human telomerase reverse transcriptase (hTERT) gene and susceptibility to gastric cancer in a Turkish population: Hospital-based case-control study. Gene. 2016 Jul 1;585(1):84-92.
81. Trifa AP, Bănescu C, Tevet M, Bojan A, Dima D, Urian L, et al. TERT rs2736100 A>C SNP and JAK2 46/1 haplotype significantly contribute to the occurrence of JAK2 V617F and CALR mutated myeloproliferative neoplasms - a multicentric study on 529 patients. Br J Haematol. 2016 Jul;174(2):218-26.
82. Xing YL, Liu F, Li JF, Lin JC, Zhu GD, Li M, et al. Case-Control Study on Impact of the Telomerase Reverse Transcriptase Gene Polymorphism and Additional Single Nucleotide Polymorphism (SNP)- SNP Interaction on Non-Small Cell Lung Cancers Risk in Chinese Han Population. J Clin Lab Anal. 2016 Nov;30(6):1071-1077.
83. Wang X, Ma KW, Chi LM, Cui JW, Jin LN, Hu JF, et al. Combining Telomerase Reverse Transcriptase Genetic Variant rs2736100 with Epidemiologic Factors in the Prediction of Lung Cancer Susceptibility. J Cancer. 2016 Apr 28;7(7):846-53.
84. Ge MH, Shi M, An CM, Yang WJ, Nie XL, Zhang J, et al. Functional evaluation of TERT-CLPTM1L genetic variants associated with susceptibility of papillary thyroid carcinoma. Sci Rep. 2016 May 17;6:26037.
85. Li XT, Xu X, Fang JL, Wang L, Mu YC, Zhang P, et al. Rs2853677 modulates Snail1 binding to the TERT enhancer and affects lung adenocarcinoma susceptibility. Oncotarget. 2016 Jun 21;7(25):37825-37838.
86. Oztas E, Kara H, Kara ZP, Aydogan MU, Uras C, Ozhan G. Association Between Human Telomerase Reverse Transcriptase Gene Variations and Risk of Developing Breast Cancer. Genet Test Mol Biomarkers. 2016 Aug;20(8):459-64.
87. Gong L, Xu Y, Hu YQ, Ding QJ, Yi CH, Huang W, et al. hTERT gene polymorphism correlates with the risk and the prognosis of thyroid cancer. Cancer Biomark. 2016 Jul 8;17(2):195-204.
88. Shiraishi K, Okada Y, Takahashi A, Kamatani Y, Momozawa Y, Ashikawa K, et al. Association of variations in HLA class II and other loci with susceptibility to EGFR-mutated lung adenocarcinoma. Nat Commun. 2016 Aug 9;7:12451
89. Dahlström J, Liu TT, Yuan XT, Saft L, Ghaderi M, Wei YB, et al. TERT rs2736100 genotypes are associated with differential risk of myeloproliferative neoplasms in Swedish and Chinese male patient populations. Ann Hematol. 2016 Oct;95(11):1825-32
90. Duan XL, Cao W, Wang LJ, Liu SD, Liu Z, Zhang BL, et al. Genetic variants in TERT are associated with risk of gastric cancer in a Chinese Han population. Oncotarget. 2016 Dec 13;7(50):82727-82732.
91. Dong J, Cheng Y, Zhu M, Wen Y, Wang C, Wang YZ, et al. Fine mapping of chromosome 5p15.33 identifies novel lung cancer susceptibility loci in Han Chinese. Int J Cancer. 2017 Aug 1;141(3):447-456.
92. Yuan XT, Cheng GH, Yu JY, Zheng SZ, Sun C, Sun Q, et al. The TERT promoter mutation incidence is modified by germline TERT rs2736098 and rs2736100 polymorphisms in hepatocellular carcinoma. Oncotarget. 2017 Apr 4;8(14):23120-23129.
93. Zhang J, Ju H, Gao JR, Jiao XL, Lu Y. Polymorphisms in human telomerase reverse transcriptase (hTERT) gene, gene- gene and gene-smoking interaction with susceptibility to gastric cancer in Chinese Han population. Oncotarget. 2017 Mar 21;8(12):20235-20243.
94. Xiao XY, He WB. Genetic polymorphisms in the TERT-CLPTM1L region and lung cancer susceptibility in Chinese males. Oncol Lett. 2017 Aug;14(2):1588-1594.
95. Chiang YH, Chang YC, Lin HC, Huang L, Cheng CC, Wang WT, et al. Germline variations at JAK2, TERT, HBS1L-MYB and MECOM and the risk of myeloproliferative neoplasms in Taiwanese population. Oncotarget. 2017 Jul 12;8(44):76204-76213.
96. Ye G, Tan N, Meng CY, Li JJ, Jing L, Yan MD, et al. Genetic variations in TERC and TERT genes are associated with lung cancer risk in a Chinese Han population. Oncotarget. 2017 Nov 6;8(66):110145-110152.
97. Aydin M, Sümbül AT, Hilaloğullari GC, Bayram S. Genetic polymorphisms in human telomerase reverse transcriptase (hTERT) gene polymorphisms do not associated with breast cancer in patients in a turkish population: hospital-based case-control study. Cell Mol Biol (Noisy-le-grand). 2018 Feb 28;64(3):108-115.

98. Li CY, Wang XN, Li YF, Zhang XW, Sun MB, Liu SY, et al. Genetic polymorphisms in the TERT gene and susceptibility to non-small cell lung cancer in a Chinese Han population. Cancer Manag Res. 2018 Jun 11;10:1487-1495.
99. Bagherpour M, Gharibzad K, Rassi H. Association of CDH1 and TERT Single-Nucleotide Polymorphisms with Susceptibility to Familial Breast Cancer Risk. Monoclon Antib Immunodiagn Immunother. 2018 Dec;37(6):239-244.
100. Li Y, Xiang C, Shen N, Deng LY, Luo X, Yuan PH, et al. Functional polymorphisms on chromosome 5p15.33 disturb telomere biology and confer the risk of non-small cell lung cancer in Chinese population. Mol Carcinog. 2019 Jun;58(6):913-921.
101. Zhang NS, Zheng Y, Liu J, Lei TS, Xu YY, Yang M. Genetic variations associated with telomere length confer risk of gastric cardia adenocarcinoma. Gastric Cancer. 2019 Nov;22(6):1089-1099
102. Bhat GR, Bhat A, Verma S, Sethi I, Shah R, Sharma V, et al. Association of newly identified genetic variant rs2853677 of TERT with non-small cell lung cancer and leukemia in population of Jammu and Kashmir, India. BMC Cancer. 2019 May 24;19(1):493.
103. Matsuguma M, Yujiri T, Yamamoto K, Kajimura Y, Tokunaga Y, Tanaka M, et al. TERT and JAK2 polymorphisms define genetic predisposition to myeloproliferative neoplasms in Japanese patients. Int J Hematol. 2019 Dec;110(6):690-698.
104. Yoo SS, Kang HG, Choi JE, Hong MJ, Do SK, Lee JH, et al. The effect of susceptibility variants, identified in never-smoking female lung cancer cases, on male smokers. Korean J Intern Med. 2020 Jul;35(4):929-935.
105. Arimura-Omori M, Kiyohara C, Yanagihara T, Yamamoto Y, Ogata-Suetsugu S, Harada E, et al. Association between Telomere-Related Polymorphisms and the Risk of IPF and COPD as a Precursor Lesion of Lung Cancer: Findings from the Fukuoka Tobacco-Related Lung Disease (FOLD) Registry. Asian Pac J Cancer Prev. 2020 Mar 1;21(3):667-673.
106. Ma LL, Fan YX, Han ZC, Su Y, Chen R, Xu R, et al. Genetic variations associated with telomere length affect the risk of gastric carcinoma. Medicine (Baltimore). 2020 Jun 5;99(23):e20551.
107. Arshad A Pandith 1, Zahoor A Wani 1, Iqbal Qasim 1, Dil Afroze 1, Usma Manzoor 1, Ina Amin, et al. Association of strong risk of hTERT gene polymorphic variants to malignant glioma and its prognostic implications with respect to different histological types and survival of glioma cases. J Gene Med. 2020 Nov;22(11):e3260
108. Mandour I, Mohamed-Hussein SA, Essam R, El-Hossainy MA. Study of genetic variants in chromosome 5p15.33 region in non-smoker lung cancer patients. Adv Respir Med. 2020;88(6):485-494.
109. Wang HJ, Yang H, Feng T, Geng TT, Chen C, Jin TB. Effects of TERT gene polymorphism and environmental factor interactions on lung cancer risk in the Xi'an Han population. Int J Clin Exp Med 2016;9(2):4200-4210.
