# Supplementary material for: Cumulative Evidence for Relationships Between Multiple Variants in the TERT and CLPTM1L Region and Risk of Cancer and Non-Cancer Disease
Source: Front Oncol. 2022 Jun 30;12:946039. doi: 10.3389/fonc.2022.946039 (PMC9279858; doi:10.3389/fonc.2022.946039)
Supplement: Supplementary file 5 [file DataSheet_5.pdf]

Supplementary Table 4: Variants in *TERT-CLPTM1L* genes showing no relation to cancer or non-cancerous risk in meta-analyses in additive model

Gene	Variant	Alleles[a]	Disease	Ethnicity	MAF[b]	Number Evaluation				Risk of Meta-Analysis		Heterogeneity		Power(%)	the value of Power(%) if the MAF is 0.2	the value of power(%) if the MAF is 0.1
						Datasets	Cases	Controls	Sample size	OR (95%CI)	P_{value}	I^2(%)	P_Q			
TERT	MNS16A	L/S	Breast cancer	Caucasian	0.2260	4	2452	2554	5006	1.065 (0.845-1.341)	0.595	73.6	0.010	83.84	80.64	56.91
TERT	rs13167280	G/A	Breast cancer	Caucasian	0.7312	3	5057	5702	10759	0.963 (0.888-1.043)	0.349	0.0	0.681	99.11	98.20	86.54
TERT	rs2075786	A/G	Breast cancer	Caucasian	0.6460	3	5057	5702	10759	0.996 (0.942-1.054)	0.902	0.0	0.716	99.69	98.20	86.54
TERT	rs2735940	A/G	Breast cancer	Overall	0.5282	6	5514	6640	12154	0.978 (0.913-1.048)	0.534	31.4	0.212	99.93	98.86	89.26
TERT	rs2735940	A/G	Breast cancer	Caucasian	0.5324	5	5446	6049	11495	1.006 (0.957-1.059)	0.803	83.6	< 0.001	99.92	98.78	88.89
TERT	rs2736100	C/A	Breast cancer	Caucasian	0.4677	4	1262	1118	2380	1.062 (0.946-1.192)	0.309	0.0	0.976	69.84	52.65	33.42
TERT	rs2736109	G/A	Breast cancer	Overall	0.4663	8	9457	12824	22281	0.987 (0.922-1.056)	0.704	59.1	0.017	99.99	99.98	98.72
TERT	rs2736109	G/A	Breast cancer	Caucasian	0.4696	7	9130	12257	21387	0.995 (0.957-1.035)	0.809	64.8	0.009	99.99	99.98	98.45
TERT	rs2736109	G/A	Breast cancer	Asian	0.3343	1	327	567	894	1.023 (0.841-1.246)	0.817	NA	NA	22.59	17.80	12.20
TERT	rs2853669	T/C	Breast cancer	Caucasian	0.4864	10	8635	10358	18993	1.006 (0.942-1.074)	0.870	41.6	0.080	99.99	99.96	97.96
TERT	rs2853677	G/A	Breast cancer	Caucasian	0.4124	3	5057	5702	10759	1.036 (0.980-1.095)	0.211	0.0	0.780	99.84	98.20	86.54
TERT	rs2853690	G/T	Breast cancer	Caucasian	0.1496	3	5057	5702	10759	0.977 (0.905-1.054)	0.541	0.0	0.847	95.26	98.20	86.54
TERT	rs7712562	A/C	Breast cancer	Caucasian	0.2791	3	5057	5702	10759	1.035 (0.927-1.156)	0.540	50.4	0.133	99.48	98.20	86.54
TERT	rs2735940	A/G	Gastric cancer	Overall	0.3234	3	641	1528	2169	1.302 (0.689-2.460)	0.416	94.1	< 0.001	38.76	30.33	19.36
TERT	rs2735940	A/G	Gastric cancer	Asian	0.3150	2	537	1319	1856	1.748 (0.894-3.419)	0.103	93.8	< 0.001	33.11	26.22	16.98
TERT	rs2736098	C/T	Hepatocellular carcinoma	Asian	0.3453	3	846	867	1713	1.211 (0.948-1.548)	0.125	65.1	0.057	49.67	38.19	24.06
TERT	rs4246742	T/A	Lung cancer	Asian	0.6252	3	3305	3720	7025	1.133 (0.875-1.467)	0.343	82.0	0.004	97.07	90.65	69.77
TERT/CLPTM1L	rs4635969	G/A	Pancreatic cancer	Caucasian	0.2057	3	2591	5383	7974	1.026 (0.943-1.117)	0.547	36.3	0.208	83.47	82.73	59.24

[a]Major alleles (reference)/Minor alleles.

[b]Frequency of minor allele in controls.
