# Supplementary material for: Cumulative Evidence for Relationships Between Multiple Variants in the TERT and CLPTM1L Region and Risk of Cancer and Non-Cancer Disease
Source: Front Oncol. 2022 Jun 30;12:946039. doi: 10.3389/fonc.2022.946039 (PMC9279858; doi:10.3389/fonc.2022.946039)
Supplement: Supplementary file 6 [file DataSheet_6.pdf]

Supplementary Table 5. Analyses of expression quantitative trait locus (eQTL) in significant variants associated with risk of cancer and non-neoplastic diseases

Variant	Gene	Tissue	*P* value	Effect Size
rs2736100	TERT	Skin-Not Sun Exposed (Suprapubic)	4.8×10^{-5}	0.23
rs2853676	TERT	Skin-Sun Exposed (Lower Leg)	2.0×10^{-6}	0.28
	TERT	Skin-Not Sun Exposed (Suprapubic)	3.9×10^{-6}	0.29
rs2853677	TERT	Skin-Sun Exposed (Lower Leg)	1.6×10^{-5}	0.24
	TERT	Skin-Not Sun Exposed (Suprapubic)	2.6×10^{-5}	0.21
rs31489	CLPTM1L	Esophagus - Mucosa	7.10×10^{-12}	-0.29
	CLPTM1L	Skin - Not Sun Exposed (Suprapubic)	2.30×10^{-08}	-0.11
	CLPTM1L	Stomach	8.80×10^{-07}	0.11
	CLPTM1L	Skin - Sun Exposed (Lower leg)	2.2×10^{-06}	-0.097
	CLPTM1L	Artery - Tibial	2.8×10^{-05}	0.075
rs401681	CLPTM1L	Esophagus - Mucosa	6.10×10^{-14}	-0.31
	CLPTM1L	Skin - Not Sun Exposed (Suprapubic)	3.60×10^{-12}	-0.14
	CLPTM1L	Skin - Sun Exposed (Lower leg)	3.30×10^{-09}	-0.12
	CLPTM1L	Stomach	3.1×10^{-06}	0.097
rs402710	CLPTM1L	Esophagus - Mucosa	4.00×10^{-11}	-0.29
	CLPTM1L	Skin - Not Sun Exposed (Suprapubic)	4.10×10^{-08}	-0.12
	CLPTM1L	Skin - Sun Exposed (Lower leg)	2.80×10^{-06}	-0.1
rs465498	CLPTM1L	Esophagus - Mucosa	1.50×10^{-14}	-0.31
	CLPTM1L	Skin - Not Sun Exposed (Suprapubic)	5.90×10^{-12}	-0.13
	CLPTM1L	Skin - Sun Exposed (Lower leg)	1.40×10^{-08}	-0.11
	CLPTM1L	Stomach	6.2×10^{-06}	0.093
rs4975616	CLPTM1L	Esophagus - Mucosa	4.30×10^{-13}	0.3
	CLPTM1L	Skin - Not Sun Exposed (Suprapubic)	6.30×10^{-10}	0.12
	CLPTM1L	Skin - Sun Exposed (Lower leg)	7.40×10^{-07}	0.1
	CLPTM1L	Stomach	5.3×10^{-06}	-0.096

Data source: Genotype-Tissue Expression (GTEx) Project.
