# Supplementary material for: Cumulative Evidence for Relationships Between Multiple Variants in the TERT and CLPTM1L Region and Risk of Cancer and Non-Cancer Disease
Source: Front Oncol. 2022 Jun 30;12:946039. doi: 10.3389/fonc.2022.946039 (PMC9279858; doi:10.3389/fonc.2022.946039)
Supplement: Supplementary file 4 [file DataSheet_4.pdf]

Supplementary Table S3: Associations between variants in the TERT-CLPTM1L region associated with risk of human disease in meta-analysis under additive model.

ID	Gene	Variant	Alleles[a]	Disease	Disease type	Ethnicity	MAF[b]	Number Evaluation			Risk of Meta-Analysis		Heterogeneity		Venice Criteria[c]	Venice Criteria Grade	Amount of Evidence		Replication	Protection from Bias		Reason for bias Exemption	Initial study influence		Deviation from HWE	P_{egger}	P_{begger}	P_{excess}	Low OR	FPRP values[d]	Credibility of Evidence[e]
								Studies	Cases	Controls	OR (95%CI)	P_{value}	I^2 (%)	P_Q			N_{minor}	Grade		Grade	Reasons for Bias		OR (95%CI)	P_{value}							
				**Bladder cancer**																											
1	TERT	rs2736098	C/T	Bladder cancer		Overall	0.3214	5	1863	3381	**1.193 (1.085-1.313)**	< 0.001	47.8	0.104	ABA	Moderate	2935	A	B	A	No	NA	1.210 (1.084-1.349)	0.001	No	0.560	0.806	1.000	No	0.006	Strong
						Caucasian	0.2534	2	948	1649	1.132 (0.977-1.311)	< 0.001	0.0	0.886																	
						Asian	0.3591	3	915	1732	**1.240 (1.094-1.405)**	0.001	70.4	0.033																	
2	TERT	rs2736100	C/A	Bladder cancer		Overall	0.5597	4	1638	3141	**0.883 (0.803-0.970)**	0.01	0.0	0.507	ABC	Weak	4401	A	A	C	Low OR	No	0.889 (0.795-0.995)	0.04	NA	0.793	0.308	1.000	Yes	0.152	Weak
						Caucasian	0.5122	2	948	1649	0.924 (0.812-1.051)	0.228	9.0	0.294																	
						Asian	0.5912	2	690	1492	**0.837 (0.728-0.961)**	0.012	0.0	0.674																	
3	CLPTM1L	rs401681	C/T	Bladder cancer		Overall	0.3615	4	1555	2500	**0.852 (0.771-0.941)**	0.002	0.0	0.757	AAA	Strong	2766	A	A	A	No	NA	0.851 (0.754-0.961)	0.009	No	0.398	1.000	1.000	No	0.029	Strong
						Caucasian	0.6859	1	498	588	0.874 (0.716-1.018)	0.078	NA	NA																	
						Asian	0.3376	3	1057	1912	**0.851 (0.754-0.961)**	< 0.001	0.0	0.554																	
4	CLPTM1L	rs402710	C/T	Bladder cancer		Overall	0.3236	3	1454	2179	**0.863 (0.772-0.965)**	0.01	0.0	0.635	AAA	Strong	1795	A	A	A	No	NA	0.840 (0.733-0.962)	0.012	NA	0.233	1.000	1.000	No	0.156	Strong
						Caucasian	0.3179	2	948	1649	0.897 (0.781-1.031)	0.127	0.0	0.810																	
						Asian	0.3349	1	506	530	**0.804 (0.667-0.969)**	0.022	NA	NA																	
				**Breast cancer**																											
5	TERT	MNS16A	L/S	Breast cancer		Caucasian	0.2260	4	2452	2554	1.065 (0.845-1.341)	0.595	73.6	0.010																	
6	TERT	rs13167280	G/A	Breast cancer		Caucasian	0.7312	3	5057	5702	0.963 (0.888-1.043)	0.349	0.0	0.681																	
7	TERT	rs2075786	A/G	Breast cancer		Caucasian	0.6460	3	5057	5702	0.996 (0.942-1.054)	0.902	0.0	0.716																	
8	TERT	rs2735940	A/G	Breast cancer		Overall	0.5282	6	5514	6640	0.978 (0.913-1.048)	0.534	31.4	0.212																	
						Caucasian	0.5324	5	5446	6049	1.006 (0.957-1.059)	0.803	83.6	< 0.001																	
						Asian	0.4629	1	68	591	2.688 (1.816-3.980)	< 0.001	NA	NA																	
9	TERT	rs2736098	C/T	Breast cancer		Caucasian	0.3004	6	4591	5159	**0.834 (0.714-0.973)**	0.021	72.3	0.003	ACC	Weak	5704	A	C	C	First study, small study	No	0.776 (0.602-1.000)	0.05	NA	0.027	0.144	1.000	No	0.286	Weak
10	TERT	rs2736100	C/A	Breast cancer		Caucasian	0.4677	4	1262	1118	1.062 (0.946-1.192)	0.309	0.0	0.976																	
11	TERT	rs2736109	G/A	Breast cancer		Overall	0.4663	8	9457	12824	0.987 (0.922-1.056)	0.704	59.1	0.017																	
						Caucasian	0.4696	7	9130	12257	0.995 (0.957-1.035)	0.809	64.8	0.009																	
						Asian	0.3343	1	327	567	1.023 (0.841-1.246)	0.817	NA	NA																	
12	TERT	rs2853669	T/C	Breast cancer		Caucasian	0.4864	10	8635	10358	1.006 (0.942-1.074)	0.870	41.6	0.080																	
13	TERT	rs2853677	G/A	Breast cancer		Caucasian	0.4124	3	5057	5702	1.036 (0.980-1.095)	0.211	0.0	0.780																	
14	TERT	rs2853690	G/T	Breast cancer		Caucasian	0.1496	3	5057	5702	0.977 (0.905-1.054)	0.541	0.0	0.847																	
15	TERT	rs7712562	A/C	Breast cancer		Caucasian	0.2791	3	5057	5702	1.035 (0.927-1.156)	0.540	50.4	0.133																	
				**Colorectal cancer**																											
16	TERT	rs2736100	C/A	Colorectal cancer		Caucasian	0.4980	10	19050	21500	**1.070 (1.040-1.102)**	< 0.001	0.0	0.700	AAC	Weak	40220	A	A	C	Low OR	No	1.072 (1.039-1.106)	< 0.001	No	0.298	1.000	0.725	Yes	< 0.001	Moderate
				**Esophageal squamous cell carcinoma**																											
17	TERT	rs2736100	C/A	Esophageal cancer		Asian	0.6055	3	2098	2150	**0.724 (0.664-0.789)**	< 0.001	0.0	0.779	AAA	Strong	4805	A	A	A	No	NA	0.725 (0.655-0.803)	< 0.001	NA	0.330	0.296	1.000	No	< 0.001	Strong
18	TERT	rs2853691	A/G	Esophageal cancer		Asian	0.2486	3	2098	2150	**1.304 (1.149-1.479)**	< 0.001	40.6	0.186	ABA	Moderate	2331	A	B	A	No	NA	1.227 (1.095-1.375)	< 0.001	NA	0.827	1.000	0.399	No	0.001	Strong
19	CLPTM1L	rs401681	C/T	Esophageal cancer		Asian	0.3386	3	1742	1856	**0.867 (0.784-0.958)**	0.005	16.2	0.303	AAA	Strong	2296	A	A	A	No	NA	0.807 (0.706-0.924)	0.002	NA	0.499	1.000	1.000	No	0.088	Strong
20	CLPTM1L	rs451360	C/A	Esophageal cancer		Asian	0.1258	3	2098	2150	**0.700 (0.610-0.904)**	< 0.001	0.0	0.979	BAA	Moderate	925	B	A	A	No	NA	0.701 (0.597-0.823)	< 0.001	NA	0.449	0.296	1.000	No	0.156	Moderate
				**Gastric cancer**																											
21	TERT	rs10069690	C/T	Gastric cancer		Asian	0.1816	4	2470	2236	**1.317 (1.193-1.454)**	< 0.001	28.5	0.241	ABA	Moderate	1969	A	B	A	No	NA	1.317 (1.193-1.454)	< 0.001	No	0.304	0.308	0.331	No	< 0.001	Strong
22	TERT	rs2735940	A/G	Gastric cancer		Overall	0.3234	3	641	1528	1.302 (0.689-2.460)	0.416	94.1	< 0.001																	
						Caucasian	0.3684	1	104	209	0.695 (0.485-0.996)	0.047	NA	NA																	
						Asian	0.3150	2	537	1319	1.748 (0.894-3.419)	0.103	93.8	< 0.001																	
23	TERT	rs2736100	C/A	Gastric cancer		Overall	0.6270	4	1843	2195	**0.751 (0.568-0.993)**	0.044	85.1	< 0.001	ACC	Weak	4771	A	C	C	HWE	No	0.673 (0.539-0.839)	< 0.001	Yes	0.244	0.734	0.611	No	0.514	Weak
						Caucasian	0.4880	1	104	209	**0.604 (0.429-0.850)**	0.004	NA	NA																	
						Asian	0.6418	3	1739	1986	0.795 (0.580-1.091)	0.155	87.7	< 0.001																	
24	TERT	rs2853676	T/C	Gastric cancer		Asian	0.7990	4	2182	2400	**0.675 (0.484-0.942)**	0.021	89.5	< 0.001	ACC	Weak	7551	A	C	C	Small study	No	0.803 (0.724-0.890)	< 0.001	No	0.064	0.734	0.448	No	0.428	Weak
				**Glioma**																											
25	TERT	rs2736100	C/A	Glioma		Overall	0.5046	8	5750	8379	**0.746 (0.666-0.835)**	< 0.001	77.0	< 0.001	AAA	Strong	12734	A	A*	A	No	Identified by GWAS	0.748 (0.654-0.855)	< 0.001	No	0.234	0.386	1.000	No	< 0.001	Strong
						Caucasian	0.4934	6	4668	7112	**0.796 (0.747-0.847)**	< 0.001	25.0	0.246																	
						Asian	0.6575	2	1082	1267	**0.493 (0.255-0.954)**	0.036	91.4	0.001																	
26	TERT	rs2853676	T/C	Glioma		Overall	0.7477	7	5832	8143	**0.784 (0.743-0.828)**	< 0.001	0.0	0.504	AAA	Strong	20069	A	A	A	Publication bias	Identified by GWAS	0.787 (0.739-0.838)	< 0.001	No	0.257	0.072	1.000	No	< 0.001	Strong
						Caucasian	0.7263	5	4423	6623	**0.794 (0.748-0.843)**	< 0.001	0.0	0.670																	
						Asian	0.8401	2	1409	1520	**0.737 (0.645-0.843)**	< 0.001	49.5	0.159																	
				**Hepatocellular carcinoma**																											
27	TERT	rs2736098	C/T	Hepatocellular carcinoma		Asian	0.3453	3	846	867	1.211 (0.948-1.548)	0.125	65.1	0.057																	
				**Idiopathic pulmonary fibrosis (IPF)**																											
28	TERT	rs2736100	C/A	Idiopathic pulmonary fibrosis (IPF)		Asian	0.5898	3	397	1848	**1.788 (1.508-2.120)**	< 0.001	0.0	0.910	AAA	Strong	2751	A	A	A	No	Identified by GWAS	1.732 (1.386-2.165)	< 0.001	NA	0.397	0.296	1.000	No	< 0.001	Strong
				**Lung cancer**																											
29	TERT	rs2242652	G/A	Lung cancer	Miscellany	Asian	0.1650	3	3631	4013	**1.168 (1.054-1.294)**	0.003	0.0	0.562	AAA	Strong	1774	A	A	A	No	NA	1.250 (1.051-1.486)	0.012	NA	0.570	0.296	0.558	No	0.053	Strong
30	TERT	rs2736098	C/T	Lung cancer	Miscellany	Overall	0.3199	10	5350	6115	**1.212 (1.121-1.310)**	< 0.001	47.0	0.049	ABA	Moderate	7834	A	B	A	No	NA	1.232 (1.162-1.306)	< 0.001	No	0.237	0.152	1.000	No	< 0.001	Strong
						Caucasian	0.2259	1	874	1061	1.158 (0.989-1.358)	0.069	NA	NA																	
						Asian	0.3350	9	4476	5054	**1.221 (1.118-1.334)**	< 0.001	52.3	0.237																	
	TERT	rs2736098	C/T	Lung cancer	NSCLC (Adenocarcinoma)	Asian	0.3502	3	1131	2203	**1.401 (1.261-1.557)**	< 0.001	0.0	0.740																	
	TERT	rs2736098	C/T	Lung cancer	NSCLC (Squamous cell carcinoma)	Asian	0.3502	3	556	2203	1.098 (0.954-1.263)	0.193	0.0	0.691																	
31	TERT	rs2736100	C/A	Lung cancer	Miscellany	Overall	0.5024	27	33918	35844	**0.856 (0.788-0.931)**	< 0.001	90.9	< 0.001	AAA	Strong	66825	A	A*	A	Small study	Identified by GWAS	0.854 (0.782-0.932)	< 0.001	No	0.067	0.802	0.122	No	0.005	Strong
						Caucasian	0.4927	5	15861	16515	**0.874 (0.845-0.903)**	< 0.001	0.0	0.754																	
						Asian	0.5907	21	18017	19289	**0.847 (0.758-0.948)**	0.004	91.9	< 0.001																	
						African	0.0972	1	40	40	1.061 (0.541-2.078)	0.864	NA	NA																	
	TERT	rs2736100	C/A	Lung cancer	NSCLC	Overall	0.6028	10	7730	19200	**0.786 (0.729-0.848)**	< 0.001	62.8	0.004																	
						Caucasian	0.4801	1	200	553	**0.714 (0.566-0.901)**	0.005	NA	NA																	
						Asian	0.6065	10	7530	18647	**0.791 (0.731-0.857)**	< 0.001	65.7	0.003																	
	TERT	rs2736100	C/A	Lung cancer	NSCLC (Adenocarcinoma)	Overall	0.4962	17	8506	30944	**0.801 (0.694-0.926)**	0.003	92.7	< 0.001																	
						Caucasian	0.4801	1	112	553	**0.675 (0.503-0.905)**	0.009	NA	NA																	
						Asian	0.4965	16	8394	30391	**0.809 (0.698-0.939)**	0.005	93.0	< 0.001																	
	TERT	rs2736100	C/A	Lung cancer	NSCLC (Squamous cell carcinoma)	Overall	0.5645	4	1065	2717	0.920 (0.827-1.025)	0.13	0.0	0.458																	
						Caucasian	0.4801	1	48	553	0.741 (0.485-1.132)	0.166	NA	NA																	
						Asian	0.5861	3	1017	2159	0.934 (0.836-1.044)	0.231	0.0	0.467																	
32	TERT	rs2853677	G/A	Lung cancer	Miscellany	Asian	0.5881	3	1123	1340	**0.791 (0.704-0.890)**	< 0.001	1.5	0.362	AAA	Strong	2835	A	A	A	Small study	Identified by GWAS	0.797 (0.693-0.917)	0.002	No	0.074	0.296	1.000	No	0.002	Strong
	TERT	rs2853677	G/A	Lung cancer	NSCLC	Asian	0.6796	3	1085	1236	**0.761 (0.672-0.861)**	< 0.001	0.0	0.639																	
	TERT	rs2853677	G/A	Lung cancer	NSCLC (Adenocarcinoma)	Asian	0.3255	5	4443	16528	1.062 (0.850-1.326)	0.596	94.1	< 0.001																	
33	CLPTM1L	rs31489	C/A	Lung cancer	Miscellany	Overall	0.3546	7	13850	14390	**0.860 (0.813-0.909)**	< 0.001	44.7	0.093	AAA	Strong	19290	A	A*	A	No	Identified by GWAS	0.851 (0.795-0.911)	< 0.001	NA	0.304	0.368	1.000	No	< 0.001	Strong
						Caucasian	0.4068	4	11361	11546	**0.866 (0.817-0.919)**	< 0.001	51.8	< 0.001																	
						Asian	0.1496	3	2489	2844	**0.833 (0.708-0.979)**	0.027	51.7	0.126																	
	CLPTM1L	rs31489	C/A	Lung cancer	NSCLC	Overall	0.2938	3	1725	2343	0.842 (0.603-1.070)	0.159	70.7	0.033																	
						Caucasian	0.4098	1	1154	1137	0.884 (0.785-0.995)	0.042	NA	NA																	
						Asian	0.1845	2	571	1206	0.826 (0.505-1.349)	0.444	82.3	0.017																	
34	CLPTM1L	rs401681	C/T	Lung cancer	Miscellany	Overall	0.3600	17	11280	12390	**0.885 (0.840-0.932)**	< 0.001	35.5	0.073	AAA	Strong	16224	A	A*	A	Low OR	Identified by GWAS	0.887 (0.837-0.940)	< 0.001	No	0.735	0.902	0.287	Yes	< 0.001	Strong
						Caucasian	0.4284	3	4306	4503	**0.874 (0.822-0.929)**	< 0.001	0.0	0.926																	
						Asian	0.3812	14	6974	7887	**0.891 (0.830-0.957)**	0.002	46.7	0.028																	
	CLPTM1L	rs401681	C/T	Lung cancer	NSCLC	Overall	0.3527	10	10109	13800	**0.846 (0.790-0.906)**	< 0.001	48.0	0.044																	
						Caucasian	0.4425	2	4470	2575	**0.855 (0.797-0.917)**	< 0.001	0.0	0.649																	
						Asian	0.3321	8	5639	11225	**0.853 (0.773-0.940)**	0.001	56.7	0.024																	
	CLPTM1L	rs401681	C/T	Lung cancer	NSCLC (Adenocarcinoma)	Overall	0.4083	7	2783	4780	0.950 (0.886-1.019)	0.15	0.0	0.792																	
						Caucasian	0.4425	2	1559	2575	0.939 (0.858-1.027)	0.167	57.1	0.127																	
						Asian	0.3666	5	1224	2205	0.968 (0.865-1.082)	0.565	0.0	0.960																	
	CLPTM1L	rs401681	C/T	Lung cancer	NSCLC (Squamous cell carcinoma)	Overall	0.4083	7	2283	4780	**0.857 (0.787-0.934)**	< 0.001	4.4	0.393																	
						Caucasian	0.4425	2	1819	2575	**0.847 (0.774-0.927)**	< 0.001	0.0	0.510																	
						Asian	0.3666	5	464	2205	0.876 (0.743-1.033)	0.117	23.5	0.264																	
	CLPTM1L	rs401681	C/T	Lung cancer	Small cell carcinoma	Overall	0.3781	4	1283	3153	0.908 (0.822-1.003)	0.058	0.0	0.712																	
						Caucasian	0.6483	1	1028	1438	0.889 (0.793-0.997)	0.045	NA	NA																	
						Asian	0.3225	3	255	1715	0.968 (0.792-1.183)	0.747	0.0	0.655																	
35	CLPTM1L	rs402710	C/T	Lung cancer	Miscellany	Overall	0.3339	16	20135	25250	**0.857 (0.832-0.883)**	< 0.001	0.0	0.873	AAA	Strong	25531	A	A	A	Small study	Identified by GWAS	0.858 (0.833-0.885)	< 0.001	No	0.099	0.101	0.110	No	< 0.001	Strong
						Caucasian	0.3515	3	11190	14329	**0.857 (0.822-0.893)**	< 0.001	0.0	0.685																	
						Asian	0.3168	11	8945	10921	**0.858 (0.821-0.896)**	< 0.001	0.0	0.747																	
	CLPTM1L	rs402710	C/T	Lung cancer	NSCLC	Asian	0.3204	4	5640	10521	**0.832 (0.785-0.882)**	< 0.001	0.0	0.421																	
	CLPTM1L	rs402710	C/T	Lung cancer	NSCLC (Adenocarcinoma)	Asian	0.3026	2	2099	3329	**0.868 (0.796-0.947)**	0.002	0.0	0.587																	
36	TERT	rs4246742	T/A	Lung cancer	Miscellany	Asian	0.6252	3	3305	3720	1.133 (0.875-1.467)	0.343	82.0	0.004																	
37	CLPTM1L	rs465498	A/G	Lung cancer	Miscellany	Asian	0.1701	4	8948	9805	**0.765 (0.723-0.810)**	< 0.001	0.0	0.880	AAA	Strong	5746	A	A	A	No	NA	0.768 (0.719-0.821)	< 0.001	NA	0.930	0.734	0.468	No	< 0.001	Strong
38	TERT/CLPTM1L	rs4975616	G/A	Lung cancer	Miscellany	Overall	0.6387	7	11300	8873	**1.159 (1.108-1.212)**	< 0.001	32.7	0.146	AAA	Strong	25885	A	A*	A	No	Identified by GWAS	1.158 (1.101-1.219)	< 0.001	NA	0.213	0.592	0.717	No	< 0.001	Strong
						Caucasian	0.5896	4	9553	7213	**1.159 (1.105-1.216)**	< 0.001	56.1	0.044																	
						Asian	0.8517	3	1747	1660	**1.155 (1.006-1.326)**	0.041	0.0	0.577																	
	TERT/CLPTM1L	rs4975616	G/A	Lung cancer	NSCLC	Overall	0.6029	3	1554	1890	1.234 (0.979-1.556)	0.075	66.1	0.052																	
						Caucasian	0.5731	2	1354	1690	1.279 (0.929-1.761)	0.132	82.8	0.016																	
						Asian	0.8550	1	200	200	1.110 (0.743-1.659)	0.609	NA	NA																	
				**Myeloproliferative neoplasms (MPNs)**																											
39	TERT	rs2736100	C/A	Myeloproliferative neoplasms (MPNs)	Miscellany	Overall	0.5846	7	2436	19204	**0.586 (0.538-0.637)**	< 0.001	0.0	0.848	AAA	Strong	24318	A	A	A	No	NA	0.577 (0.527-0.633)	< 0.001	NA	0.643	0.230	1.000	No	< 0.001	Strong
						Caucasian	0.4925	4	1956	1791	**0.589 (0.532-0.654)**	< 0.001	0.0	0.552																	
						Asian	0.5940	3	480	17413	**0.578 (0.498-0.670)**	< 0.001	0.0	0.764																	
	TERT	rs2736100	C/A	Myeloproliferative neoplasms (MPNs)	Essential thrombocythemia (ET)	Overall	0.5909	4	792	18145	**0.589 (0.522-0.665)**	< 0.001	0.0	0.707																	
						Caucasian	0.5228	2	552	833	**0.561 (0.481-0.655)**	< 0.001	0.0	0.536																	
						Asian	0.5942	2	240	17312	**0.637 (0.525-0.774)**	< 0.001	0.0	0.970																	
	TERT	rs2736100	C/A	Myeloproliferative neoplasms (MPNs)	Polycythemia vera (PV)	Overall	0.5909	4	499	18145	**0.521 (0.449-0.604)**	< 0.001	46.7	0.131																	
						Caucasian	0.5228	2	393	833	**0.562 (0.473-0.669)**	< 0.001	0.0	0.745																	
						Asian	0.5942	2	106	17312	**0.422 (0.318-0.562)**	< 0.001	64.7	0.092																	
	TERT	rs2736100	C/A	Myeloproliferative neoplasms (MPNs)	Primary myelofibrosis (PMF)	Overall	0.5909	4	201	18145	**0.575 (0.463-0.713)**	< 0.001	0.0	0.472																	
						Caucasian	0.5228	2	168	833	**0.538 (0.423-0.686)**	< 0.001	2.3	0.312																	
						Asian	0.5942	2	33	17312	0.758 (0.464-1.237)	0.267	0.0	0.908																	
				**Pancreatic cancer**																											
40	CLPTM1L	rs401681	C/T	Pancreatic cancer		Caucasian	0.4398	3	2591	5383	**1.173 (1.097-1.255)**	< 0.001	0.0	0.974	AAA	Strong	7088	A	A	A	No	NA	1.174 (1.097-1.258)	< 0.001	NA	0.752	1.000	1.000	No	< 0.001	Strong
41	TERT/CLPTM1L	rs4635969	G/A	Pancreatic cancer		Caucasian	0.2057	3	2591	5383	1.026 (0.943-1.117)	0.547	36.3	0.208																	
				**Skin cancer (melanoma)**																											
42	CLPTM1L	rs401681	C/T	Skin cancer	melanoma	Caucasian	0.4415	3	1188	1603	**1.285 (1.120-1.414)**	< 0.001	23.5	0.270	AAA	Strong	2532	A	A	A	No	NA	1.207 (1.051-1.386)	0.008	NA	0.103	0.296	1.000	No	0.002	Strong
				**Thyroid cancer**																											
43	TERT	rs2736100	C/A	Thyroid cancer		Asian	0.5969	4	2752	2752	**0.762 (0.657-0.884)**	< 0.001	72.2	0.013	ACC	Weak	6033	A	C	C	Small study, excess of significant findings	No	0.719 (0.629-0.820)	< 0.001	No	0.074	0.734	0.062	No	0.007	Moderate

Note: OR, odds ratio; A, adenine; T, thymine; G, guanine; C, cytosine; NSCLC: non-small cell lung cancer; NA: Not Available.
HWE:=Yes: Significance lost excluding studies with controls violating HWE; No: Significance was not lost excluding the study with controls violating HWE;
Low OR = Low magnitude of the association (i.e. 0.87<OR<1.15)
Publication bias= Possible existence of potential publication bias (Begg's test, p<0.10)
Small study (Sample size<100) = Possible existence of potential small study effect (modified Egger's test, p<0.10).
An excess of significant findings based on the excess significance test. (p<0.10 is usually considered evidence for significant evidence of an excess of significant findings.)

[a]Major alleles (reference)/ minor alleles.

[b]Frequency of minor allele in controls.

[c]Strength of epidemiological evidence based on the Venice criteria.

[d]The prior probability of FPRP is 0.05, and the FPRP level of noteworthiness is 0.20.

[e]Degree of epidemiological credibility based on the combination of results from Venice guidelines and FPRP tests.
