# Supplementary material for: Cumulative Evidence for Relationships Between Multiple Variants in the TERT and CLPTM1L Region and Risk of Cancer and Non-Cancer Disease
Source: Front Oncol. 2022 Jun 30;12:946039. doi: 10.3389/fonc.2022.946039 (PMC9279858; doi:10.3389/fonc.2022.946039)
Supplement: Supplementary file 7 [file DataSheet_7.pdf]

Supplementary Table 6. Linkage Disequilibrium (r^2) among the significant variants in the *TERT-CLPTM1L* genes.

SNP Pair[1]	Asian[2]	European[2]	African[2]
rs10069690 & rs2242652	0.9166	0.6409	0.0657
rs10069690 & rs2736098	0.0067	0.0124	0.0045
rs10069690 & rs2736100	0.2693	0.2812	0.1816
rs10069690 & rs2853676	0.2337	0.0905	0.0718
rs10069690 & rs2853677	0.1285	0.0360	0.0376
rs10069690 & rs2853691	0.0047	0.0526	0.0021
rs2242652 & rs2736098	0.0049	0.3800	0.0014
rs2242652 & rs2736100	0.2709	0.1864	0.1387
rs2242652 & rs2853676	0.2453	0.1117	0.3249
rs2242652 & rs2853677	0.1286	0.0633	0.2350
rs2242652 & rs2853691	0.0574	0.1523	0.0068
rs2736098 & rs2736100	0.2416	0.0937	0.0493
rs2736098 & rs2853676	0.0025	0.0002	0.1597
rs2736098 & rs2853677	0.3070	0.1504	0.0032
rs2736098 & rs2853691	0.0012	0.0012	0.0033
rs2736100 & rs2853676	0.2057	0.2475	0.1062
rs2736100 & rs2853677	0.5789	0.4352	0.2031
rs2736100 & rs2853691	0.0110	0.0104	0.0063
rs2853676 & rs2853677	0.2392	0.3317	0.2192
rs2853676 & rs2853691	0.0096	0.0024	0.0106
rs2853677 & rs2853691	0.0031	0.0002	0.0000
rs31489 & rs401681	0.4785	0.8145	0.6131
rs31489 & rs402710	0.4634	0.6624	0.8376
rs31489 & rs465498	0.9373	0.8516	0.6572
rs401681 & rs402710	0.9371	0.6624	0.5961
rs401681 & rs465498	0.4839	0.9601	0.9332
rs402710 & rs465498	0.4692	0.5959	0.6345
rs31489 & rs10069690	0.0005	0.0112	0.0809
rs31489 & rs2242652	0.0004	0.0335	0.0295
rs31489 & rs2736098	0.0379	0.1400	0.0463
rs31489 & rs2736100	0.0091	0.0129	0.0300
rs31489 & rs2853676	0.0058	0.0051	0.1224
rs31489 & rs2853677	0.0001	0.0372	0.0693
rs31489 & rs2853691	0.0117	0.0065	0.0029
rs401681 & rs10069690	0.0147	0.0250	0.0752
rs401681 & rs2242652	0.0139	0.0649	0.0552
rs401681 & rs2736098	0.0857	0.1696	0.0809
rs401681 & rs2736100	0.0458	0.0039	0.0023
rs401681 & rs2853676	0.0009	0.0050	0.2054
rs401681 & rs2853677	0.0179	0.0145	0.0471
rs401681 & rs2853691	0.0185	0.0114	0.0004
rs402710 & rs10069690	0.014	0.0013	0.0611
rs402710 & rs2242652	0.0133	0.0080	0.0287
rs402710 & rs2736098	0.0853	0.0996	0.0483
rs402710 & rs2736100	0.0426	0.0179	0.0197
rs402710 & rs2853676	0.0007	0.0274	0.1252
rs402710 & rs2853677	0.0174	0.0098	0.0729
rs402710 & rs2853691	0.0003	0.0142	0.0025

rs465498 & rs10069690	0.0002	0.0236	0.0841
rs465498 & rs2242652	0.0000	0.0624	0.0528
rs465498 & rs2736098	0.0404	0.1696	0.0748
rs465498 & rs2736100	0.0053	0.0044	0.0051
rs465498 & rs2853676	0.0083	0.0050	0.1896
rs465498 & rs2853677	0.0000	0.0155	0.0483
rs465498 & rs2853691	0.0060	0.0105	0.0000

Note: NA = not applicable.
[1]Data source: ldlink.nci.nih.gov.
[2]Asians, European, and African were selected from five major population categories provided by the databases.
